# Curcumin Encapsulated in Crosslinked Cyclodextrin Nanoparticles Enables Immediate Inhibition of Cell Growth and Efficient Killing of Cancer Cells

**DOI:** 10.3390/nano11020489

**Published:** 2021-02-15

**Authors:** Karin Möller, Beth Macaulay, Thomas Bein

**Affiliations:** Department of Chemistry and Center for NanoScience, University of Munich (LMU), Butenandtstrasse 5–13, 81377 Munich, Germany; beth.macaulay@gmail.com

**Keywords:** crosslinked cyclodextrin nanoparticles, curcumin, label-free imaging, anti-cancer, drug delivery, kinetic response profiles, IC_50_

## Abstract

The efficiency of anti-cancer drugs is commonly determined by endpoint assays after extended incubation times, often after days. Here we demonstrate that curcumin encapsulated in crosslinked cyclodextrin nanoparticles (CD-NP) acts extremely rapidly on cell metabolism resulting in an immediate and complete inhibition of cell growth and in efficient cancer-cell killing only few hours after incubation. This early onset of anti-cancer action was discovered by live-cell high-throughput fluorescence microscopy using an environmental stage. To date, only very few examples of covalently crosslinked nanoscale CD-based (CD-NP) drug carriers exist. Crosslinking cyclodextrins enables the adsorption of unusually high payloads of hydrophobic curcumin (762 µg CC/mg CD-NP) reflecting a molar ratio of 2.3:1 curcumin to cyclodextrin. We have investigated the effect of CD-NP encapsulated curcumin (CD-CC-NP) in comparison to free, DMSO-derived curcumin nanoparticles (CC-NP) on 4 different cell lines. Very short incubations times as low as 1 h were applied and cell responses after medium change were subsequently followed over two days. We show that cell proliferation is inhibited nearly immediately in all cell lines and that a cell- and concentration dependent cancer-cell killing occurs. Anti-cancer effects were similar with free and encapsulated curcumin, however, encapsulation in CD-NP drastically extends the long-term photostability and anti-cancer activity of curcumin. Curcumin-sensitivity is highest in HeLa cells reaching up to 90% cell death under these conditions. Sensitivity decreased from HeLa to T24 to MDA MB-231 cells. Strikingly, the immortalized non-cancerous cell line MCF-10A was robust against curcumin concentrations that were highly toxic to the other cell lines. Our results underline the potential of curcumin as gentle and yet effective natural anti-cancer agent when delivered solvent-free in stabilizing and biocompatible drug carriers such as CD-NP that enable efficient cellular delivery.

## 1. Introduction

Many potent anti-cancer drugs are too hydrophobic for direct pharmaceutical use [1]. As a result, suitable drug formulation is key for successful cancer treatment. Drug delivery systems face demanding issues, they have to be biocompatible and biodegradable, non-toxic and of limited final size, to name a few, and they still need to be able to carry a sufficient drug load. In this context, cyclodextrin (CD) and CD derivatives have been explored as drug delivery agents since several decades based on their favorable solubilizing capabilities for hydrophobic drugs, their high absorption capacity and their low toxicity. In fact, plain supramolecular CD assemblies were applied as active pharmaceutical ingredients (APIs) against a number of diseases, e.g., by affecting the cholesterol homeostasis in leukemic cells [2,3]. Mostly, however, they are used as carriers to improve the bioavailability of many APIs by forming noncovalent inclusion complexes that are being explored in a number of clinical trials [4,5]. β-Cyclodextrin was first approved for systemic applications by the United States FDA as constituent of the antifungal (itraconazole) medication Sporanox in 1997 [6]. Today, new promising CD-based drugs are on the market, such as Kyprolis (Carfilzomib) against relapsed multiple myeloma, using the highly soluble sulfobutylether β-cyclodextrin derivative [7,8]. Also, the first clinical trials for siRNA delivery in 2008 were based on CD-containing polymers [6,9]. CDs are FDA-approved for oral, intravenous as well as subcutaneous applications [10]. Today, there are over 40 pharmaceutical products on the market using CD as carrier material [11].

CDs are cyclic oligosaccharides composed of 6, 7 or 8 D-glucopyranose subunits which constitute hydrophobic cavities with diameters of about 5, 7 and 9 Å. They are able to readily absorb numerous APIs, most of them being poorly soluble in aqueous media and/or are highly toxic [12]. Thus, cyclodextrins are valued for improving the solubility of many drugs, for minimizing detrimental side effects by forming host-guest inclusion compounds and furthermore for stabilizing drugs against premature degradation, thus consequently increasing their bioavailability. CD-based formulations are prepared using different strategies [13], usually based on forming a spontaneous self-assembly of drug and CD units into aggregates [14], the assembly process often being aided by polymers [15] or by polymer-functionalized CD derivatives (e.g., through ionotropic gelation [16,17]), or by attaching cyclodextrins to nanoparticle (NP) scaffolds such as Au-NP or silica NP [6,10,14,18,19,20,21]. Supramolecular assemblies with high surface areas were developed as CD metal organic frameworks (CD-MOF) [22]. However, being assembled via metal ions forming coordinative bonds to the hydroxyls of the cyclodextrin units, they are not stable in aqueous solutions at neutral pH.

In contrast, direct covalent crosslinking of the CD building blocks can substantially improve the carrier stability and avoid the exposure to potentially toxic metal ions. Moreover, such CD nanoparticles could potentially achieve higher loading capacities (within the network constituting the NP) and allow dual delivery of components within a single host. Reports on crosslinked CD nanoparticles are scarce. Suitable crosslinking agents such as hexamethylene diisocyanate, diphenyl- or dimethyl-carbonates, pyromellitic dianhydride or carbonyldiimidazole were reported to generate crosslinked CD bulk materials, which were fabricated into particles usually larger than 400 nm in size by crushing the dried polymer materials [23,24]. The group of Trotta has conducted extensive research on the synthesis and application of these ‘nanosponge’ materials and has demonstrated their safety for oral administration in in vivo studies [25]. In contrast, the bottom-up preparation of stable, 100–200 nm colloidal CD nanoparticles was achieved by crosslinking sulfobutylether-CD with hexamethylene diisocyanate [26]. Very small crosslinked CD-particles of ca. 30 and 80 nm were prepared using epichlorhydrin, and envisioned as carriers for pulmonary applications [27,28]. Promising nanoparticles of similar and even smaller size were achieved using succinyl-functionalized β-CD through coupling with lysine via amide-bonds, which were then used in immunotherapy applications [29].

Our group has recently reported on a new synthesis of small β-cyclodextrin nanoparticles sized about 200 nm by crosslinking with the rigid spacer tetrafluoroterephthalonitrile (TFTN) [30]. This TFTN linker was previously utilized in the synthesis of CD bulk materials which showed very good properties for micropollutant absorption [31,32]. Here, we have improved our nanoparticle synthesis with respect to higher yields and exploit the amphiphilic character of these biocompatible carriers for solubilizing large quantities of hydrophobic curcumin.

Curcumin (CC), a naturally occurring polyphenol in turmeric roots is acknowledged in numerous reports for showing a number of health benefits, ranging from being an anti-oxidant, anti-inflammatory, inhibitor of amyloid fibrillation, potent anti-carcinogenic and even anti-metastatic agent. The multi-faceted health benefits of curcumin were recently showcased in a special issue on curcumin including several reviews concerning its role in cancer applications [33,34,35,36]. Despite providing promising results against colorectal, pancreatic and breast cancer in clinical trials, its medical application is still hampered in part due to the extremely low solubility and limited stability of curcumin. When applied in its pure form, curcumin is usually dissolved in DMSO, ethanol or acetone. However, the toxicity of these solvents and the instability of unprotected curcumin has prompted the exploration of nano-carrier formulations, especially for anti-cancer applications. Delivery approaches for CC using liposomes, polymers, micelles, dopamine-stabilized fructose as well as the inclusion in cyclodextrins have been reported [37,38,39,40,41,42]. Disregarding the large number of carefully conducted in vitro and in vivo studies documenting health benefits of curcumin, critical comments were raised, warning that curcumin is a “pan-assay interference compound” (PAINS), showing unspecific protein binding leading to false results in drug screening assays [43,44,45]. However, these viewpoints are not shared by experts who recognize the potential of curcumin and value its cytotoxic properties against many cancer cell lines as an opportunity, especially when its poor bioavailability is overcome by delivery platforms that ensure high solubility, long-term stability and allow for high payloads of curcumin [46,47,48].

Here, we demonstrate that small crosslinked cyclodextrin nanoparticles (CD-NP) can function as a promising carrier for curcumin to address all these critical aspects. We highlight the immediate and dramatic impact of the NP-packaged curcumin on cell proliferation and demonstrate its efficient anti-cancer activity when delivered protected, solvent-free and highly concentrated in these CD-NP. We obtained similar anti-cancer effects also with free, DMSO-derived curcumin nanoparticles CC-NP, however, we show that the inherent limited stability of free curcumin is dramatically enhanced upon adsorption and encapsulation in our cyclodextrin nanoparticles. The discovery of the fast onset of cytotoxic action of curcumin was facilitated by applying label-free, live-cell imaging using high-throughput fluorescence microscopy equipped with an environmental stage. The cell response following curcumin exposure was tracked over extended time using high-content screening. Short primary incubation times of 1 to 3 h were chosen to approach realistic delivery conditions and were evaluated on four different cell lines. We show that the cancer-cell lines HeLa, MDA MB-231 and T24 are strongly affected by curcumin while the non-cancerous cell line MCF-10A is very resistant, surviving even high curcumin concentrations.

## 2. Results and Discussion

### 2.1. Synthesis and Characterization of Crosslinked Cyclodextrin Nanoparticles CD-NP

**Figure 1:**
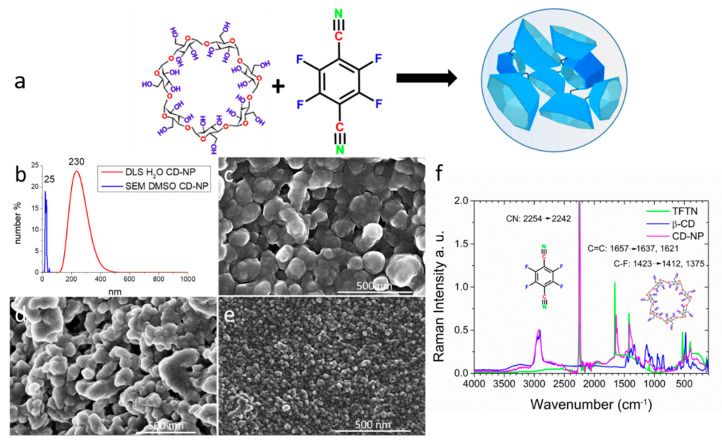
(**a**): reaction scheme of cyclodextrin and TFTN to form CD-NP. (**b**) Particle size distribution of CD-NP as determined by DLS in an aqueous solution (red, PDI = 0.26) contrasted to the size distribution of the identical sample as determined from SEM micrographs (blue) when stirred in DMSO, data taken from SEM in (**e**). (**c**,**d**) are typical SEM pictures made from an aqueous (**c**) or ethanolic (**d**) CD-NP solution showing agglomerated particles of sizes similar to those measured by DLS. (**f**) FT-Raman spectra of the precursor CD (blue), TFTN (green) and the final CD-NP (purple). Frequency shifts of TFTN in CD-NP as compared to the precursors are indicated.

The nanoparticle synthesis was performed by covalently coupling β-cyclodextrin units via the rigid spacer TFTN in order to create particles with a high absorption capacity that conceptually could exceed the inherent cavity space of the individual CD units. This linker is further equipped with nitrile groups that are ready for additional functionalization via click reactions. We used an optimized synthesis route as reported before by our group by combining β-CD, TFTN, K_2_CO_3_ and CTAB and PEG2000 in DMSO [30] and increased the overall yields to more than 60% (for a detailed synthesis procedure and characterization, see the Supporting Information, also Figures S2 and S3). FT-Raman spectroscopy indicates a successful reaction between the TFTN linker and the CD units. All prominent vibrations of the TFTN linker are significantly shifted or have developed shoulders in the crosslinked CD-NP upon bond-formation (see Figure 1f). Particles with a hydrodynamic size between 200 to 230 nm were identified by Dynamic Light Scattering (DLS) in aqueous solution. However, when freeze dried samples were dispersed either in water, ethanol or DMSO and, after drying, were analyzed by SEM it was found that the particle size is highly dependent on the solubility of the particles in the respective media. Agglomeration to a particle size resembling the original DLS data is observed in aqueous and ethanolic solutions, while a dispersion in DMSO shows much smaller particles sized between 20 to 30 nm (see Figure 1).

Nitrogen sorption measurements performed with freeze dried CD-NP samples resulted in surface areas of 30–90 m^2^/g (see Figure S3, Supplementary Information). These values are rather moderate when compared to reports of 35 to 263 m^2^/g obtained on similarly cross-linked CD-bulk materials [32]. We assume that the wall structure of our small nanoparticles is more flexible and easily folded upon exposure to high vacuum as applied during the sorption measurements. Nevertheless, this is not detrimental to very high absorption capacities in aqueous solution as demonstrated below.

### 2.2. Curcumin (CC) Absorption in Cyclodextrin Nanoparticles: CD-CC-NP

The absorption of curcumin in our CD-NP was performed using freshly made DMSO/curcumin stock solutions. To 1 mg/mL aqueous CD-NP suspension we added increasing amounts of the CC stock solution, calculated to represent molar ratios of 1:1, 1:2 or 1:3 CD:CC, which were incubated for 15 min. The separation of non-absorbed curcumin was performed by repeated centrifugation and washing and the concentration of the collected supernatants was determined by UV-Vis measurements. A rapid and nearly complete uptake of the offered curcumin was observed in all cases, revealing an extraordinarily high sorption capacity of CD-NP for curcumin. Final loading efficiencies resulted in 85 to 90% of CC offered in solution, with the highest loading accounting for 762 µg/mg curcumin/CD or a loading capacity of 43.2 wt% (final curcumin weight/weight of total product; reflecting a molar ratio after wash of CD:CC = 1:2.34; see also Figure S4, Supplementary Information). Considering the molecular dimensions of curcumin in a β-CD-cavity, model calculations suggest that only 1 of the 2 phenolic endgroups of CC can be accommodated in a CD cavity. Indeed, maximum molar ratios of 1:0.5 to 1:1 CD:CC (amounting to 14 to 24 wt%) are generally reported when individual cyclodextrin units are complexed with curcumin [49,50,51,52,53]. Crosslinking cyclodextrin thus generates significant additional sorption capacities and allows for a curcumin loading clearly exceeding even a molar ratio of 1:2 CD-NP:CC (or 39 wt%).

**Figure 2:**
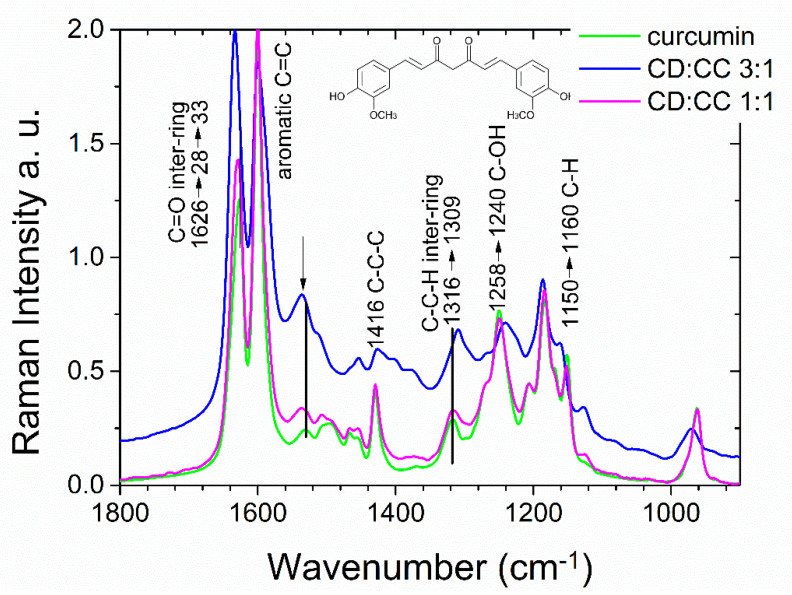
FT-Raman spectra of curcumin and curcumin adsorbed into CD-NP with molar ratios of CD:CC = 3:1 and 1:1. Intensities were normalized to the aromatic C=C Raman frequency at 1600 cm^−1^ for better comparison.

The intricate interactions between curcumin and the cyclodextrin cavities are also supported by Raman spectroscopy. Figure 2 shows Raman spectra of pure curcumin powder in comparison to curcumin adsorbed into CD-NP, using a low molar ratio of only 3:1 as well as an equimolar ratio of 1:1 CD:CC. Curcumin is a very good Raman scatterer and dominates any small cyclodextrin Raman bands in the depicted region. Analyzing the spectrum with the lowest curcumin absorption, we note that the vibrational modes of curcumin are strongly shifted, in the aromatic region as well as in the inter-ring region as indicated in the figure, suggesting a strong interaction [54,55]. At higher loading concentrations we observe a signature that resembles the spectrum of bulk curcumin.

### 2.3. Curcumin Stabilization in CD-NP

Concerns were raised regarding the application of curcumin for medical purposes, related to the known instability of this polyphenol. The photostability of curcumin is dependent on the respective solvent, as well as on pH, temperature and salt concentrations, [56,57] however, it was also reported that curcumin can be stabilized when encapsulated in micelles, gels, yeast or in cyclodextrin [55,58]. We have conducted photostability experiments to study the effect of curcumin encapsulation on its lifetime. Curcumin was either absorbed in our crosslinked CD-NP (CD-CC-NP) or directly diluted in water from the DMSO stock solution (CC-NP) at concentrations that are applied in the cell experiments described below (for DLS spectra, see Figure S5, Supplementary Information). UV-VIS measurements were performed immediately after sample preparation and were repeated after 5 days exposure to light or were monitored over 170 h when kept in the dark. These measurements showed a dramatic decay in absorbance of pure CC-NP, while encapsulated curcumin stayed nearly stable (see Figures S6–S8, Supplementary Information).

Taken together, these observations confirm that an inclusion of curcumin in cyclodextrin nanoparticles ensures a prolonged stabilization, drastically extending the lifetime of these guest molecules. Notably, long-term fluorescence stabilization was recently shown when CC was topically applied after tumor removal to prevent tumor recurrence when it was prepared as oil-nano-emulsion as opposed to CC-DMSO solutions [59]. The inclusion of CC in robust cyclodextrin-NP hosts promises not only to ensure a longer shelf-live but offers also good handling properties, enabling centrifugation, redispersion and enhanced protection in the biological environment.

### 2.4. Cell Studies: HeLa Cervical Cancer Cell Line

In the following we report on the influence of curcumin on the cell behavior of a number of different cancer cell lines as studied by high throughput microscopy using an environmental stage, maintained at 37 °C under exposure of 5% CO_2_, without movement during the entire observation time. Curcumin nanoparticles and curcumin encapsulated in crosslinked cyclodextrin nanoparticles of similar concentrations were administered with increasing concentrations to the following cancer cell lines: HeLa (cervical cancer), T24 (urinary bladder carcinoma cells) and MDA MB-231 cells (a triple negative breast cancer cell line). For comparison we included also the non-tumorigenic breast epithelial cell line MCF-10A in our study. In general, to 100 µL medium containing the respective cells seeded in 96-well plates, we added 0.6, 1.2, 1.8, 2.4 and 3 µg curcumin as CC-NP or adsorbed in CD-CC-NP. The originally offered curcumin concentrations are displayed in all graphs, however, since typically only 90% of the initially offered curcumin was absorbed in the CD-NP host we adjusted the listed IC_50_ values for these differences in the text. Cells were incubated with curcumin for 1 to 3 h (in the following termed “incubation time”), followed by a medium exchange, medium without curcumin. The cell status was further microscopically monitored for up to 3 days; this time span is referred to as “post-incubation time”.

**Figure 3:**
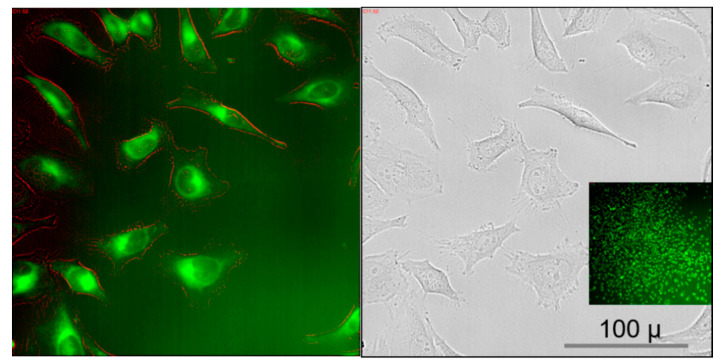
Images of HeLa cells directly after application of auto-fluorescent curcumin (10 µL of a CD-CC-NP aqueous suspension containing 1.8 µg CC were added to 100 µL well volume; red lining is due to artificial coloring for better visualization of cell morphology). The GFP channel is compared to the bright field image (magnification 40×). Inset: CC fluorescence is visible in all cells in the well, here imaged with a 10× objective.

We will describe the processes occurring in HeLa cells in greater detail, but observations made with this cell line were also made with the other cell lines. Due to the convenient auto-fluorescence of curcumin, its uptake into cells is easily followed in the microscope. This process happens nearly instantaneously in all cell lines, taking less than 1–2 min, in fact is even too fast to be captured in a movie in our setup. The green fluorescence is visible throughout the cells indicating that CC is immediately dissolved in the cell membranes and organelles, regardless if it was delivered as pure CC-NP or adsorbed in CD-CC-NP. The highest curcumin intensities were observed in areas of the endoplasmic reticulum and the nuclear envelope (see Figure 3; for more evidence see also Figure 7 and Figure S17, Supplementary Information).

The fast uptake of curcumin in the cells prompted us to study the release of curcumin from the CD-NP in different media. CD-CC-NP with a mole ratio of CD:CC 1:1 were stirred in water, DPBS and in the DMEM cell medium either with or without 10% FBS. When supernatants of these solutions were analyzed with UV-Vis or fluorescence spectroscopy, we could detect only minor amounts of curcumin in water, DPBS or in cell medium without FBS, even when samples had been stirred in these solutions for extended times. In contrast, when FBS was present in DMEM, substantial amounts, up to 36% of the total curcumin content in the CD-CC-NP was released after 15-min stirring (see Figures S9 and S10, Supplementary Information). The released curcumin showed again an absorption maximum at 425 nm in the UV-Vis spectrum, indicating that no molecular degradation had happened in the CD-NP. In contrast, curcumin extracted in the presence of FBS showed a blue-shift in the emission spectrum: while DMSO-extracted curcumin emits at 550 nm, this peak is shifted to 490 nm for curcumin extracted by FBS-exposed CD-CC-NP. This is taken as an indication for an association of curcumin with components in the FBS. We thus assume that lipids and proteins in cell membranes and cytosol are responsible for the immediate curcumin absorption.

### 2.5. Effective Suppression of Proliferation and Cell Viability after Short Term Exposure of HeLa Cells

**Figure 4:**
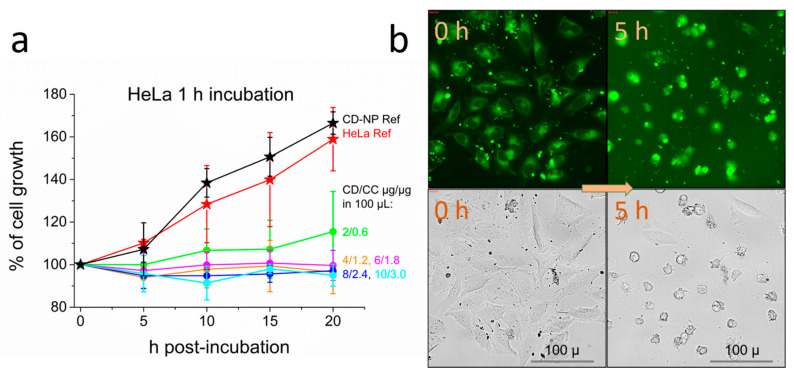
(**a**) Proliferation histogram of HeLa cells following 1 h incubation with increasing amounts of CD-CC-NP. The legend reflects the amount of the CD-NP carrier and its loading with curcumin CC as used in 100 µL volume. (**b**) GFP channel (top) and corresponding bright field images of HeLa cells (1 h incubation, CD/CC = 10/3 µg/µg, reflecting a CC concentration of 81 µM) directly after medium change (0 h) and at 5 h post-incubation.

In our systematic cell studies, HeLa cells were incubated with curcumin absorbed in cyclodextrin nanoparticles (CD-CC-NP) with a molar ratio CD:CC 1:1. Concentrations between 2 to 10 µg of these nanoparticles were added to 100 µL well volume, resulting in wells containing 0.6 to 3 µg CC. After an incubation time of 1 h we performed a medium change and continued to monitor the cells over the next 20 h post-incubation time, successively taking images of identical well areas. Three to six of these images from 3 different wells were visually inspected and evaluated by counting the total number of cells as well as by assessing the number of living and dead cells after each time step. Figure 4a shows a histogram of HeLa cell growth upon exposure to CD-CC-NP. While the untreated reference of HeLa cells grows steadily, reaching about 160% in cell number over a period of 20 h, we observed for all administered CD-CC-NP concentrations a nearly complete arrest of cell proliferation. Only a slight increase of about 10% is still visible upon adding the smallest concentration of curcumin (0.6 µg in 2 µg of CD-NP). The pure CD-NP carrier does not affect cell growth and shows a growth curve comparable to that of the untreated HeLa cells (see stars in Figure 4a: red HeLa, black CD-NP).

When comparing the images of cells directly after medium change (post-incubation time 0) and after a 5-h post-incubation time in Figure 4b, we notice dramatic morphological changes, especially in cells treated with the highest curcumin concentration (3 µg curcumin in 10 µg of CD-NP). Bright field images show that most of the cells are contracted and have died during this time, have developed microtubule spikes, blebbing and cell debris, typical for an apoptotic cell death. The strong green fluorescence of curcumin is still intense, strongest seen in the contracted dead cells and cell debris. Similar events occur upon addition of pure CC-NP under the same conditions (see Figure S11, Supplementary Information). The induction of apoptotic cell death by curcumin has been found in many different breast cancer [60,61], gastric [62] and lung cancer cell lines [63], and has also been reported for HeLa [64], T24 [65] and MDA MB-231 [66] cells as were used in this manuscript.

**Figure 5:**
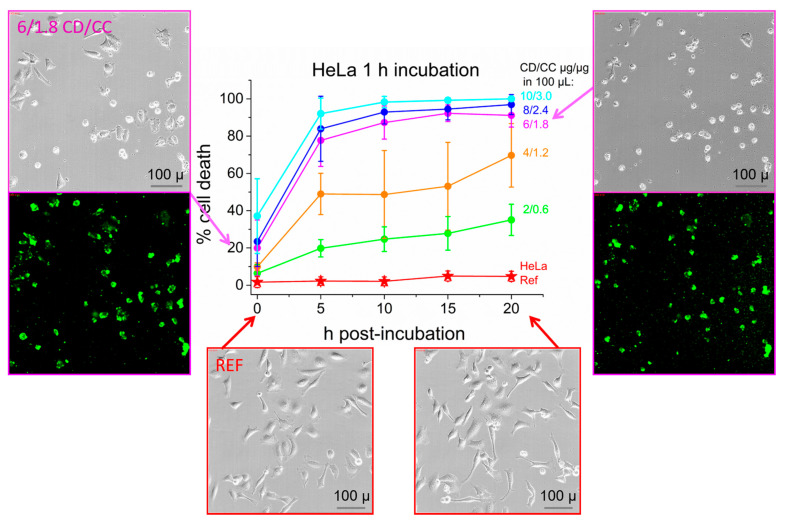
Concentration-dependent kinetic response curves displaying cell mortality in HeLa cells, incubated for 1 h with CD-CC-NP (molar ratio 1:1). Representative images after medium change (0 h, left) and at 20 h post-incubation time (right) are shown (CD/CC = 6/1.8 µg/µg). Purple color-coded images refer to the purple sample in the graph. Untreated HeLa cells are shown in red, images were taken at time points as indicated by arrows.

When we analyzed all images with respect to dead cells, we realized that CD-delivered curcumin does not only act antimitotic but shows fast anti-carcinogenic effects on HeLa cells even when they are exposed to lower concentrations as shown above. Figure 5 displays the concentration-dependent response curves with respect to the dead cell concentration. Graphs are color-coded and correspond to the graphs in Figure 4a. Partial cell death is directly visible after medium change (1h) in many wells, as seen at time point 0. For better representation we show selected images on the left of this graph, here framed in purple, indicating 1.8 µg CC per well delivered in 6 µg CD-NP. Over 20% of the cells are dead already at this time and after 5 h we encounter massive apoptosis again, even with this low concentration. After 20 h more than 90% of the cells are dead as seen in the corresponding image on the right. This killing efficiency was obtained in the concentration range between 1.8 to 3.0 µg curcumin per 100 µL while lower concentrations decreased the dead-cell count to an average value of 70% (orange curve, 1.2 µg CC in 4 µg CD-NP) and to only 39% at the lowest concentration (green curve, 0.6 mg CC in 2 mg CD-NP). Overall, an IC_50_ between 73 to 43 µM curcumin (3.0 to 1.8 µg CC/100 µL) is established within 0 to 3 h post-incubation or of 29 µM (1.2 CC/100 µL) after 5-10 h, notably after a very short incubation time of only 1 h. Similar, slightly less effective kinetic response curves were obtained when these HeLa cells were exposed to identical curcumin concentrations in form of DMSO-derived pure CC-NP (see Figures S12 and S13, Supplementary Information). These IC_50_ values reached with CD-NP-delivered curcumin can be related to doxorubicin, one of most prominent anti-cancer agents in clinical use. The Massachusetts General Hospital Listings for Drug Sensitivity in cancer present IC_50_ values for 14 different cervical cancer cell lines with doxorubicin between 0.038 and 8.53 µM, however, all cell lines were incubated for 72 h [67]. Our previous studies using similar CD-NP carriers as in this report showed an IC_50_ value of 3 µM for doxorubicin when incubated with HeLa cells for 24 h [30]. It has been noted that the IC_50_ value decreases with increasing incubation time [68]. Thus, the short incubation times and high efficiencies of curcumin in CD-NP established in this work offer promise for its use as an anti-cancer agent.

Equivalent CC concentrations as those used in Figure 5 can also be delivered to the cells using lower CD carrier concentrations when the curcumin loading is increased accordingly (CD:CC molar ratio >1:1). We have varied this ratio successively from 1:0.5, to 1:1, 1:2 and 1:3 and administered a constant CD-CC-NP dose of 2 µg/well (effective CC concentrations from 0.3 to 1.8 µg/well). Under these conditions we observed slightly slower death rates, however, an increase in incubation time could offset this delay, resulting in similar IC_50_ values as before (see Figure S13; for an example of cell counting see Figure S14, Supplementary Information).

Thus, the IC_50_ value is strongly related to the initial exposure conditions of the respective cells. In general, we find a faster/stronger cytotoxic effect upon extending the incubation time. This influence of incubation time is often neglected in cell studies, where cells are often exposed for 24 h or even longer times, which tend to drastically exceed typical circulation/cell contact times in vivo. These differences in the kinetic responses are easily obscured when only endpoint assessments are selectively performed. When we compared dead-cell-concentrations assessed after 20 h from experiments using 1, 2 or 3 h incubation times, they were all very similar, thus obscuring the early onset of cell killing (see Figure S15, Supplementary Information).

### 2.6. Transient Vacuole Formation

**Figure 6:**
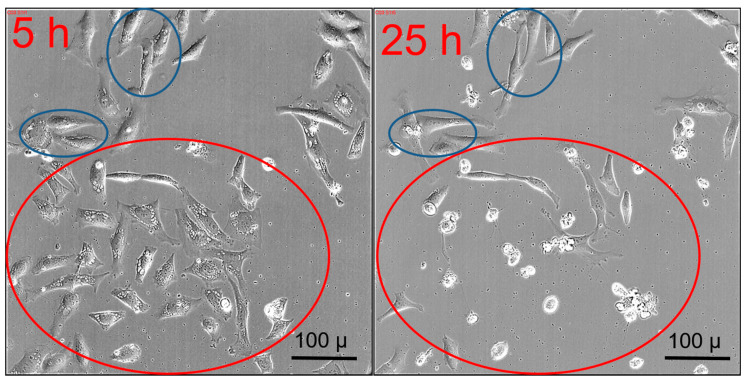
Morphological changes observed in HeLa cells at different post-incubation times after exposure to 1.2 µg curcumin (32 µM). Left: pore formation around the nuclear envelopes after 5 h that develop into dead cells after 25 h as shown on the right (highlighted in red). Highlighted in blue are cells that were able to recover.

We observed a remarkable phenomenon in HeLa cells at early stages of post-incubation with medium concentrations of curcumin. Cells that were spread out nicely on the well bottom suddenly developed changes in their internal morphology when exposed to 1.2 µg CC in CD-CC-NP (1 h incubation time, CD:CC 1:1). Large vacuoles appeared, predominantly around the nucleus and the site of the endoplasmic reticulum, an area where curcumin had accumulated mostly before. In Figure 6 we highlight specific cells where these features appeared after 5 h post-incubation. At a later stage (25 h), most of these cells have contracted and died, accompanied by massive blebbing as noticed above. Notably, some cells escaped this fate and were able to recover their normal appearance, here highlighted in blue. These changes were initialized independently of the delivery form of curcumin, either as CC-NP or absorbed in CD-NP and were observed only at intermediate post-incubation times, between 5 to 20 h, as illustrated in Figure S16, Supplementary Information. After 25 h, a time point often used for endpoint viability tests, cell fates are already determined, as some have collapsed into apoptotic cells while others have recovered. A similar ambivalent behavior of cytoplasmic vacuolization [69], causing recovery or cell death was reported before in connection with autophagy and apoptosis. Autophagy is a cell-rescue mechanism initiated by cell stress that, if successful, can degrade dysfunctional cell components such as misfolded proteins. However, if massive autophagy occurs it leads to self-destruction and apoptosis. For instance, such processes were observed in different liver cancer cells (HepG2, Huh-7) upon exposure to curcumin [70] or curcumin derivatives [71], in gastric cancer cells [62] and in heterogeneous breast cancer cells MCF-7 [72]. We observe this process also in T24 cells as shown in the following section.

**Figure 7:**
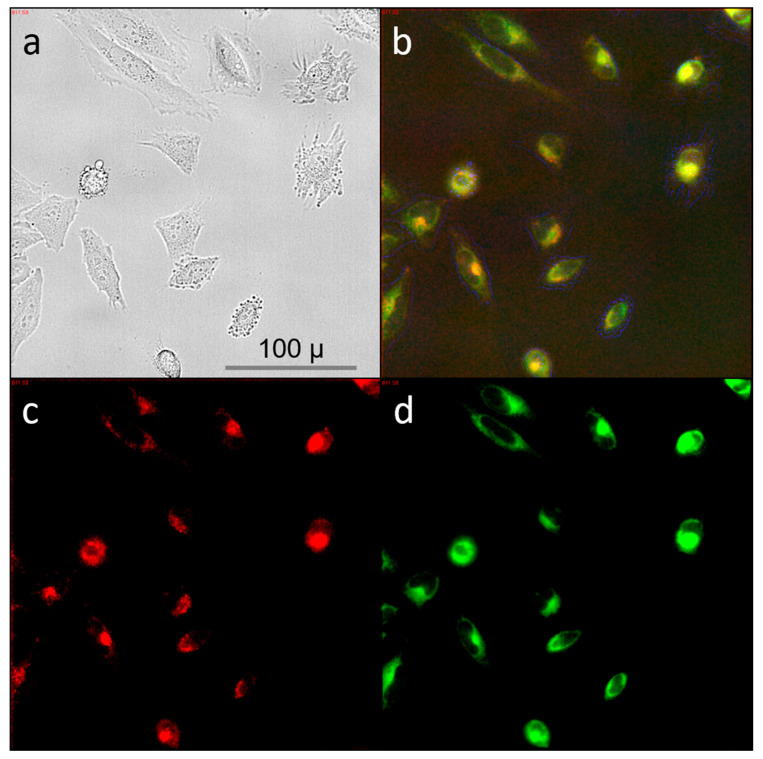
HeLa cells directly after incubation with curcumin were treated with the ER-tracker red. Shown are (**a**) bright field, (**b**) overlay of (**c**) ER-trackerRed labeled cells and (**d**) curcumin auto-fluorescing cells.

The induction of autophagy can be activated by the unfolded protein response (UPR) associated with the endoplasmic reticulum (ER) stress [73]. ER stress was reportedly stimulated by curcumin, successfully leading to apoptosis in thyroid cancer cells and in patient-derived glioblastoma cells [74,75,76]. We have labeled HeLa cells with either mitochondria tracker or ER tracker directly after application of curcumin and found a pronounced co-localization of curcumin and the endoplasmic reticulum while only a partial overlap was observed with mitochondria (see Figure 7 and Figure S17, Supplementary Information). A similar predominant localization of curcumin in the ER as opposed to mitochondria was observed upon curcumin delivery to Huh-7 liver cells [70,77]. Together with the vacuolization and apoptotic development in our cells upon curcumin application we assume that similar processes proceed in HeLa cells, where in a concentration-dependent manner either successful autophagy results in cell survival, or where in a combination of paraptosis [69] (vacuolization of components in the endoplasmic reticulum) and apoptosis cell death is induced. Stimulation of autophagy has been recently discussed as a promising therapeutic avenue in anti-cancer strategies [78]. This process clearly occurs upon curcumin exposure but can only be observed using real-time imaging due to its transient nature.

### 2.7. Cytotoxicity of Aged Curcumin Samples is Preserved if Encapsulated in CD-NP

Since we have shown above that the photostability of curcumin is markedly extended when it is encapsulated in the CD-NP carrier, we have also compared the toxicity of 5 day-aged CC-NP to 5 day-aged CD-CC-NP samples (see Figures S18 and S19, Supplementary Information). We observe a strong loss in toxicity of the aged CC-NP samples. While potent enough to arrest cell growth, these samples are not able to kill the cells. In contrast, CD-CC-NP treated cells experience similar cell cycle arrest and additional cell killing with only minor toxicity losses compared to the unaged samples.

### 2.8. Endpoint Analysis

**Figure 8:**
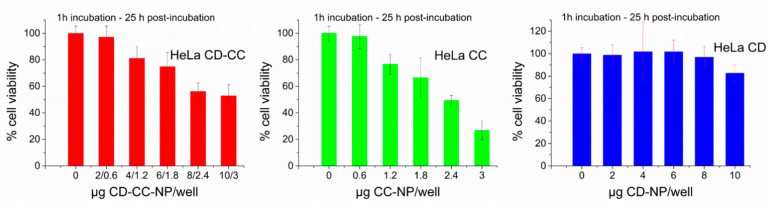
CCK-8 cell viability analysis of HeLa cells incubated with CD-CC-NP (CD:CC 1:1, red), DMSO-derived pure CC-NP (green) and the free carrier CD-NP (blue), incubation time 1 h, analyzed at 20 h post-incubation.

Traditionally, cell viability endpoint tests are performed using dye indicators based on tetrazolium salts (MTT assay). We have tested both MTT and CCK-8 indicators, the latter is based on a tetrazolium-analog forming water-soluble formazan upon reduction, which resulted in more consistent results in this study. Here, we applied the CCK-8 assay as endpoint assay in all our cell experiments. Representative results for HeLa cells are displayed in Figure 8, using an incubation time of 1 h and a 20-h post-incubation time, thus evaluating the endpoint as shown in Figure 5 above.

The viability assays establish the non-toxicity of the CD-NP carrier system over the whole concentration range, confirming our findings from image analysis. However, we do find that even CCK-8 assays generally display an apparently higher cell viability than the results from image analysis after treatment with CD-CC-NP or CC-NP, as documented in Figure 4 and Figure 5, Figures S12, S13 and S19, Supplementary Information. Discrepancies between viability assays and direct cell counting methods have been noted before, and it was recommended to use additional non-metabolic methods for verification [79,80]. Furthermore, while endpoint assays permit a relative comparison of samples when incubation and post-incubation times are carefully observed, information about cell growth, transient phenomena such as vacuolization and onset of cell death are necessarily hidden. Real-time tracking of cell development thus allows us to gain valuable information about the cell fate. With HeLa cells we could show that depending upon the exposure conditions, curcumin can be a potent anti-cancer agent, reacting efficiently after short-time exposure with regard to mitosis and cell death.

### 2.9. Cell Studies: T24 Bladder Carcinoma Cells Need Longer Incubation Times

**Figure 9:**
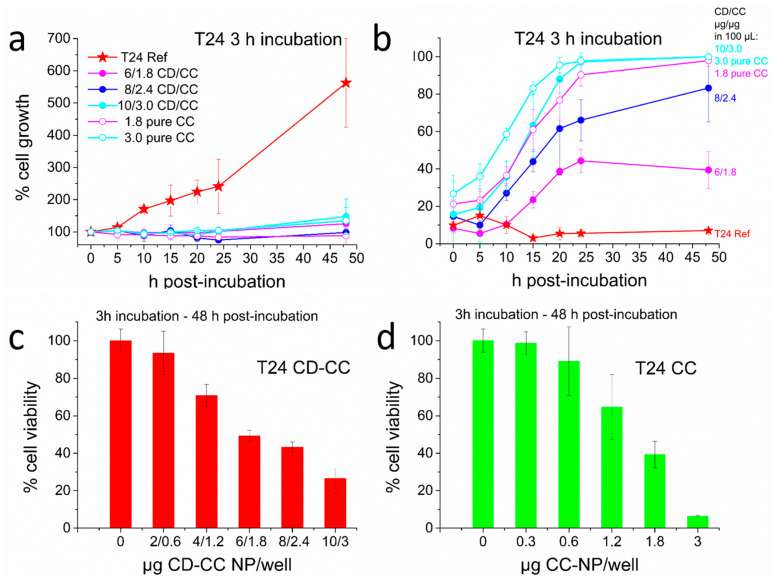
Effect of CC-NP and CD-CC-NP on T24 bladder cancer cells using a 3 h incubation, (**a**) proliferation histogram, (**b**) kinetic response curves displaying cell mortality, (**c**,**d**) corresponding CCK-8 viability assays performed after 48 h on (**c**) CD-CC-NP treated cells and (**d**) CC-NP treated cells.

Similar to the studies discussed above with HeLa cells, we also performed experiments with other cancer cell lines. Bladder cancer is commonly treated with cis-platin but treatment is often impaired by developing tumor resistance. Only scarce information exists about the activity of curcumin with respect to T24 bladder cancer cells, but a diminished cell proliferation and apoptosis was reported, caused presumably by suppression of the matrix metalloproteinase MMP pathway [65]. A combined delivery of curcumin and cis-platin (each 10 µM) was also found to increase the fraction of apoptotic cells to 30%, notably after a 24 h incubation period [81]. In our experiments, we observed a higher resistance of T24 cells towards curcumin in comparison to our results with HeLa cells. Applying again only a 1 h incubation time with samples equivalent to those used with HeLa cells, we observed suppression of cell growth starting at a concentration of 1.8 µg/well (48 µM) curcumin, but dead cells were hardly present. Cell mortality strongly increased when 3 h incubation was used with the same CC-NP (see Figure S20, Supplementary Information). For this reason, we extended the incubation time to 3 h. Now, a complete stop of cell proliferation still required the delivery of 1.8 µg CC (48 µM), but was accompanied by massive cell killing. After 20 h post-incubation, up to 97% of the cells had died when the highest concentration of 3 µg CC (81 µM) was applied as CD-CC-NP or even when only 1.8 µg CC (48 µM) were applied as CC-NP (see Figure 9a,b and Figure S21, Supplementary Information, for corresponding images). CCK-8 viability assays after 48 h reflect the situation that the pure CC-NP were slightly more effective here than the CD-CC-NP. However, these assays suggest again a higher cell viability than the one directly observed in the micrographs.

One reason for this extended survival of T24 cells might be found in the successful exocytosis of curcumin-affected cell organelles. We observed a time-dependent accumulation of auto-fluorescing particles in the cell medium when T24 cells were treated with the high concentration of 3 µg CC in CD-CC-NP (see Figure S22, Supplementary Information). Vacuolization occurred, similar to the behavior of HeLa cells, already under lower concentration of 1.8 µg CD-CC-NP after 24 h (see Figure S23, Supplementary Information).

### 2.10. Cell Studies: Triple Negative MDA MB-231 Cancer Cells need Extended Incubation Times and Higher Concentrations

The MDA MB-231 cell line is an aggressive triple negative breast cancer line prone to develop chemo-resistance against powerful therapeutics such as doxorubicin or paclitaxel. Curcumin was shown to reverse this resistance when delivered in combination with doxorubicin to MDA MB-231 cells and to increase the sensitivity of breast cancer stem cells to paclitaxel, cis-platin and mitomycin C [82,83]. We explored the cytotoxic properties of curcumin when used against MDA MB-231 cells by proceeding similarly as described above for the HeLa and T24 cell lines. Similar to the response of the T24 cell line, we observe that a short exposure to CD-CC-NP or pure CC-NP of only 1 h incubation was insufficient to show any impact on cell viability.

**Figure 10:**
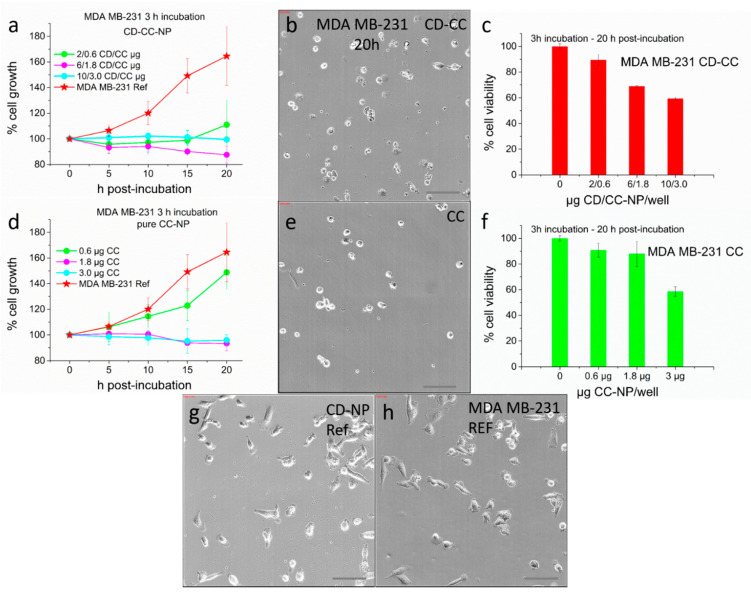
Effect of curcumin on MDA MB-231 cells after 3 h incubation followed by 20 h post-incubation: (**a**–**c**) CC delivered in CD-CC-NP, (**d**–**f**) treated with pure CC-NP. (**b**,**e**,**g**,**h**) Bright field images after 20 h post-incubation, cells treated with (**b**) 3 µg CC in CD-CC-NP, (**e**) 3 µg CC as CC-NP, (**g**) 10 µg CD-NP/well, (**h**) untreated reference, scale bar: 100 µ. (**a**,**d**) Cell proliferation obtained by cell counting, (**c**,**f**) CCK-8 assay after 20 h post-incubation.

However, a retardation of cell growth was observed even here when treated with the highest curcumin concentration encapsulated in CD-CC-NP (see Figure S24b, Supplementary Information). It required 3 h incubation to show a remarkable suppression of proliferation at the lowest concentration of 0.6 µg curcumin (16 µM), again more pronounced when delivered via CD-CC-NP (see Figure 10). Here we refrained from analyzing the microscope images using cell death counting since these cells react very sensitively to any treatment and contract immediately, making a distinction between life/dead cells susceptible to mistakes with real-time label-free analysis (see also Figure S25, Supplementary Information). However, after 20 h post-incubation we do see that proliferation of the cells is indeed substantially reduced, compared to the untreated reference or to the cells treated with the CD-NP carrier alone (Figure 10g,h). Here, the cells are spread out and clearly alive while cells exposed to curcumin appear mostly dead. The respective CCK-8 results indicate a reduction of cell viability by about 40% with the highest CC concentration, similar in CD-CC-NP and CC-NP treated cells. This concentration translates into a relatively high 73 µM solution (3 µg CC/100 µL/well). Lower IC_50_ values of 20 µM curcumin were reported for this cell line when curcumin was micellized in amphiphilic block-co-polymers with high loading capacities [48] or applied as DMSO solution [62], and it was even reduced to 10 µM curcumin under simultaneous application of an equimolar doxorubicin dose [82]. However, all cells used in these experiments were incubated for 72 h. Thus, a 40% efficacy at a relatively short exposure time of only 3 h may validate the application of curcumin against this aggressive cell line, possibly as a highly efficient addition in combination cancer therapy.

### 2.11. Cell Studies: Non-Cancerous Human Mammary Epithelial Breast Cell Line MCF-10A

**Figure 11:**
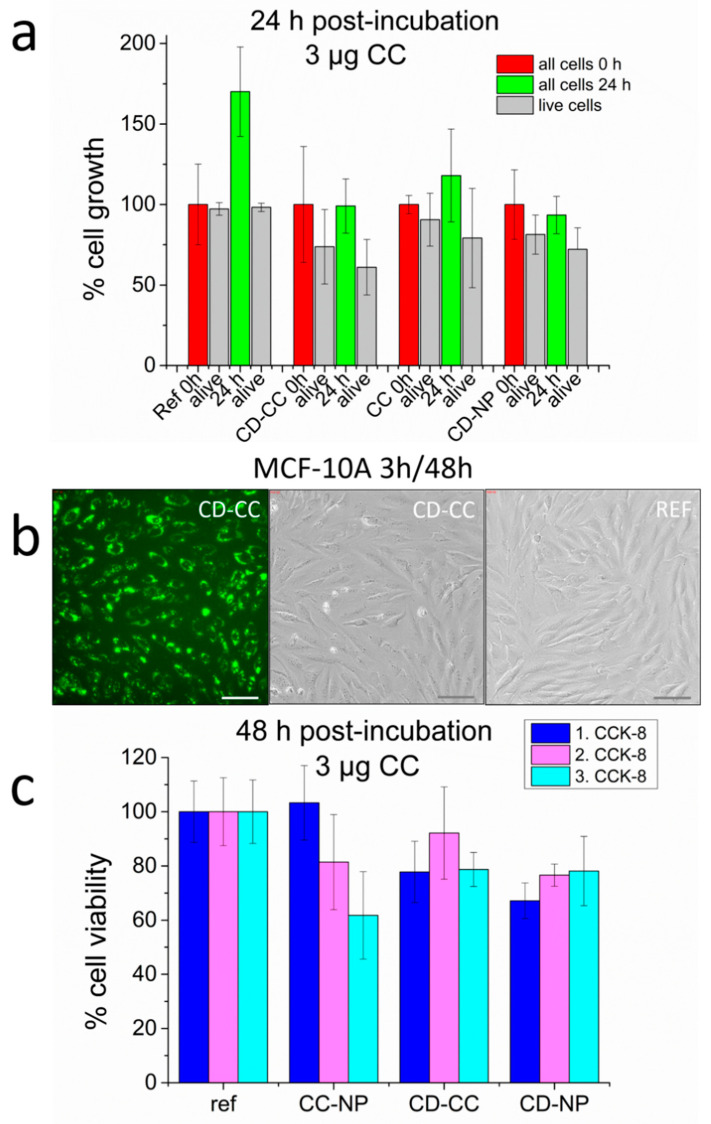
MCF-10A cell response to 3 µg/well curcumin delivery, 3 h incubation: (**a**) CC delivered as CD-CC-NP and CC-NP in comparison to pure CD-NP, cell count after 24 h post-incubation (green), including the survival rate (grey), (**b**) images after 48 h post-incubation with CD-CC-NP in comparison to untreated cells, scale bar 100 µm, (**c**) comparison of three CCK-8 assays performed after 48 h, here results for 3 µg CC/well.

Common anti-cancer treatments are usually non-discriminately aggressive against all cell lines and often induce severe side-effects. We have included a non-cancerous cell line in our studies to evaluate the cytotoxic effect of curcumin towards healthy cells. Exposure conditions to CD-CC-NP and CC-NP were set to 3 h incubation and 48 h post-incubation, the maximum values used for the cancer cell lines discussed above. In none of the four independent experiments performed did we encounter comparable mortality rates as observed for any of the other cell lines described above. In fact, even with the highest concentration of curcumin of 3 µg/well, we encountered at most a 30–40% mortality. In Figure 11a we compare the cell proliferation (cell counts) after 24 h post-incubation (green bars) to the starting conditions (after medium change, 0 h, red bars). The treatment does show a suppression of cell growth upon exposure to free CC-NP, CD-CC-NP or even CD-NP, while untreated cells grow by about 165% during this time period. However, most of the cells are still alive (grey bars). The good survival of MCF-10A cells under the highest CD-CC-NP concentration even after 48 h is seen in the images in Figure 11b. Substantial CD-CC-NP particle uptake has taken place, but cells are mostly alive. Only in one instance did we recognize even here the formation of vacuoles, only in 2 wells upon exposure to the highest CC-NP concentration, and not with a similar CD-CC-NP concentration (see Figure S26, Supplementary Information). Importantly, most of these cells experienced a recovery. This resistance of MCF-10A cells towards curcumin is further reflected in CCK-8 assays employed after 48 h and shown in Figure 11c, summarizing results of 3 independent experiments for this concentration. While the results for pure CC-NP spread over 62 to 103% viability, we measure a more consistent survival rate between 78 to 92% for CD-CC-NP and 68 to 78% for the pure CD-NP (for the complete CCK-8 assays see Figure S27, Supplementary Information).

The notable resistance of non-cancerous cells towards curcumin in contrast to its remarkable killing efficiency with respect to cancer cell lines has now been documented in several studies for a number of different cell lines. For instance, human mesenchymal stem cells (MSC) showed higher resistance to curcumin delivered in a cyclodextrin/liposomal formulation than KHOS cells (osteosarcoma, bone cancer cells) [50]. Similarly, fibroblast L929 cells were less affected than breast cancer MCF-7 cells [41], and curcumin delivered as nano-emulsion was much less toxic to non-cancerous human HEK-293T cells than to gastric (AGS), colon (HT29-ATCC, HT29-US), breast (MDA MB-231) and melanoma (B16F10) cells [59]. It also was less toxic towards WI38 lung normal fibroblast cells than towards A549 lung cancer cells when simply absorbed in α-CD [84]. Even curcumin analogs showed a lower toxicity towards normal than towards cancerous liver cells [71]. These encouraging results taken together strongly suggest to seriously consider curcumin as anti-cancer remedy, either as stand-alone or as adjuvant medication.

## 3. Outlook and Summary

Numerous articles have documented the health benefits and anticancer activities of curcumin, and excellent review articles summarize the collected knowledge about its mechanistic action, such as the binding to cancer associated targets (e.g., the nuclear factor kb (NFkB) and CX-2, both connected to tumor progression), [85,86] the inhibition of gene expression of pro-metastasis associated enzymes (e.g., the MMP matrix metalloprotease, responsible for cell invasion) [87] or the regulation of cancer-suppressive miRNAs (e.g., miRNA-22), [88,89] culminating in induction of apoptosis and inhibition of tumor cell proliferation.

Here, we complement these findings by performing cytotoxicity assessments through microscopy observations of transient cell responses towards curcumin exposure directly from the start. In vitro cell experiments represent the most basic set-up for determining the potential of a successful anti-cancer agent. Our work was thus designed to explore the minimum requirements for efficient curcumin toxicity towards cancer cell lines. Incubation times were accordingly chosen as short as possible to approach realistic in vivo conditions. By following the response of cancer cell lines directly without employing disturbing labelling procedures and by evaluating cell images by visual inspection in time, we were able to determine the cell-specific conditions for achieving cancer cell growth inhibition and the induction of apoptosis.

We present a novel carrier system based on covalently cross-linked cyclodextrin units that is able to absorb very high payloads of small hydrophobic anti-cancer agents as demonstrated here using polyphenolic curcumin. The encapsulation of curcumin in CD-NP is advantageous for a number of reasons even though comparable results were obtained with free (fresh) curcumin nanoparticles in this study: CD-nanoparticles are stable against centrifugation and redispersion, ease handling and storage requirements and simultaneously offer a pronounced solubility enhancement of the curcumin guest molecules. The nanoparticles stabilize curcumin against photodegradation and more importantly, prolong the cytotoxic potential of curcumin as shown in cell experiments using aged samples. Furthermore, by absorbing curcumin into these carriers, cell exposure to toxic (and pharmaceutically unrealistic) solvents such as DMSO is eliminated.

Our study highlights the real-time label-free microscopic cell observation as a valuable tool to assess the optimal conditions for curcumin anti-cancer activity. Cell incubation was minimized to only 1–3 h in order to approach realistic delivery conditions. Notably, this incubation time influences the value of the apparent IC_50_ value which decreases upon prolonged cell exposure. Cell responses to curcumin were directly imaged in time over periods of 24 to 48 h, enabled by a high-throughput environmental stage attached to the fluorescence microscope. The resulting cell growth and cell death histograms revealed the prompt and pronounced cytotoxic effect of curcumin, which would escape detection by the sole application of endpoint assays. We document a nearly complete killing of HeLa cells already after 5 h post-incubation, even when cells were exposed for only 1 h to curcumin before.

Bladder cancer cells T24 and breast cancer cells MDA MB-231 require longer exposure times to curcumin, but a 3 h incubation period showed successful cell killing also with these cell lines. In contrast, non-cancerous MC-10A cells display a profound resistance against curcumin, supporting the promising biocompatibility of curcumin. Importantly, cell proliferation was completely stopped in all cell lines at low concentrations and at early stages of post-incubation, an effect that has been related to the induction of a G2/M cell cycle arrest [90]. This phenomenon by itself seems advantageous for using encapsulated curcumin as anti-cancer remedy, even more so when considering curcumin’s pronounced and selective toxicity towards cancer cell lines. Furthermore, using aged curcumin samples we could show that packaging in CD-NP significantly prolongs its cytotoxic activity. In summary, we established the highest toxicity of curcumin towards HeLa cells, a pronounced cytotoxicity towards T24 cells and even a promising activity against highly resistant MDA MB-231 cells, while non-cancerous MCF-10A cells experienced a good survival rate even at the highest applied curcumin concentration.

## Supplementary Materials

The following are available online at https://www.mdpi.com/2079-4991/11/2/489/s1, Materials, synthesis and anal information; Figure S1: UV-Vis curcumin calibration curve; Figure S2: CD-NP characterization: Thermogravimetric analysis; Figure S3: CD-NP characterization: Nitrogen sorption; Figure S4: Loading efficiencies of curcumin in CD-NP; Figure S5: Particle size stability of DMSO-derived pure curcumin nanoparticles; Figures S6–S8: Photostability of DMSO-derived curcumin nanoparticles in comparison to curcumin encapsulated in CD-NP: UV-VIS spectroscopy; Figure S9: Medium-dependent release of curcumin from CD-NP; Figure S10: Fluorescence Spectroscopy of curcumin; Figure S11: Apoptotic response of HeLa cells after pure curcumin nanoparticle delivery; Figure S12: Concentration-dependent kinetic response curves upon delivery of pure curcumin nanoparticles to HeLa Cells; Figure S13: Influence of curcumin loading by changing the CD:CC molar ratio in HeLa cells; Figure S14: Example for cell counting; Figure S15: Impact of curcumin loading by changing the CD:CC molar ratio: endpoint analysis (cell counting) HeLa cells; Figure S16: Development of cytoplasmic vacuolization in HeLa cells; Figure S17: HeLa cell labeling with mitochondrial tracker CMXRos; Figure S18: Cytotoxicity of aged curcumin samples; Figure S19: Concentration-dependent curcumin activity on HeLa cells using 5 d aged samples; Figures S20–S23: Effect of curcumin on T24 cells; Figures S24 and S25: Curcumin delivery to MDA MB-231 cells; Figures S26 and S27: Curcumin delivery to MCF10A cells
