# Supplementary material for: Curcumin Encapsulated in Crosslinked Cyclodextrin Nanoparticles Enables Immediate Inhibition of Cell Growth and Efficient Killing of Cancer Cells
Source: Nanomaterials (Basel). 2021 Feb 15;11(2):489. doi: 10.3390/nano11020489 (PMC7919290; doi:10.3390/nano11020489)
Supplement: Supplementary file 1 [file nanomaterials-11-00489-s001.pdf]

Supplementary Material

Curcumin Encapsulated in Crosslinked Cyclodextrin Nanoparticles Enables Immediate Inhibition of Cell Growth and Efficient Killing of Cancer Cells

Karin Möller *, Beth Macaulay and Thomas Bein *

Department of Chemistry and Center for NanoScience, University of Munich (LMU), Munich, Butenandtstrasse 5–13, 81377 Munich, Germany; beth.macaulay@gmail.com
* Correspondence: K.Moeller@lmu.de (K.M.); bein@lmu.de (T.B.); Fax: (+49) 89-2180-77622 (T.B.)

1. Materials:

β-cyclodextrin (CD; 97%), potassium carbonate (K_2CO_3, 99.5%), polyethylene glycol 2000 (PEG2000), Pluronic F-127, cetyltrimethylammonium bromide (CTAB), dimethylsulfoxide (DMSO, anhydrous, >99.9%), ethanol (EtOH, absolute), hydrochloric acid 1 M (HCl), and curcumin (CC, >94% curcuminoid content) were all purchased from Merck, Sigma Aldrich , Darmstadt, Germany. Tetrafluoroterephtalonitrile (TFTN, 98%), 5-tamra-azide (Baseclick), Dulbecco's phosphate-buffered saline (DPBS, no calcium, no magnesium), Dulbecco's modified Eagle's medium (DMEM), Fluo-Brite DMEM ,and Hoechst 33342 trihydrochloride were all purchased from ThermoFisher Scientific, Kandel, Germany; ER-trackerRed and Mito Tracker Red CMXRos (Invitrogen), bidistilled water (Milli-Q Academic A10). Penicillin, streptomycin, and fetal bovine serum (FBS) were purchased from ThermoFisher, Schwerte, Germany . CCK-8 cell counting kits were obtained from Dojindo EU GmbH Munich, Germany.

2. Synthesis of Crosslinked CD-NP

The nanoparticle synthesis can be performed via different reaction routes, and a scanning of the reaction parameters indicated that the synthesis described here is robust with respect to variations in molar ratios of the reactants. However, it is important to use an excess of the coupling agent TFTN (close to a molar ratio of 1:3 with respect to CD). It is not necessary to work in completely anhydrous conditions, since a reaction under dry nitrogen did not result in retrievable particles. The duration of the reaction should be at least 3 h; a prolonged reaction time resulted generally in higher yields. The flocculating agents, HCl and water, need to be added slowly and without stirring. We performed the crosslinking under conventional as well as a microwave heating conditions and two representative reactions are described below:

2.1. Conventional Reaction Procedure:

We combined 200 mg β-CD (0.157 mmol), 100 mg TFTN (0.5 mmol), 300 mg K_2CO_3 (2.17 mmol), 10 mg PEG2000 (5 μmol), and 25 mg CTAB (0.07 mmol) with 7 mL anhydrous DMSO in a 40 mL polypropylene container. The container was closed and the mixture was sonicated for 15 min and subsequently heated under stirring (750 rpm) at 80 °C for 3–7 hours. The brownish suspension was cooled to room temperature. The product was flocculated by carefully adding 7 mL water and 7 mL hydrochloric acid (1 M) to the reaction mixture without stirring. The floating flocculate was collected into 2–4 centrifuge tubes by pipette, and the process was repeated until the flocculation stopped or until the reaction medium was completely converted. Subsequent centrifugation at 7830 rpm (7197 rcf) for 15 min resulted in a pale yellow solid. Extensive washing was performed (2× with 1 M HCl, 2× with water, and 2× with ethanol), using ultrasound re-dispersion and centrifugation (15 min, 7197 rcf).
The light yellow solid was finally dispersed in water with concentrations of about 4–5 mg/mL. The yield ranged from 29% to over 60%, depending on the reaction time (assuming CD:TFTN of 1:1).

2.2. Microwave Synthesis

The microwave reaction was performed using a Synthos 3000 microwave (Anton Paar, Graz, Austria) with 15 reactor slots and 1 reference, Teflon-lined 100 mL reactors, loading volume 6–60 mL each, with twice the amount as described

above. Two experiments were simultaneously prepared using either 20 mg PEG 2000 or 20 mg Pluronic F-127. The reaction was performed with two reaction vessels, applying a heating ramp of 1 min to 80 °C with a maximum power of 1200 W. The reaction was performed for 1 h at this temperature. A reference reactor contained 600 mg K_2CO_3 dissolved in 15 mL DMSO. Flocculation and washing were performed as above. The yield was 20% with PEG 2000 and 40% using Pluronic F-127.

3. Preparation of CC-NP:

Fresh curcumin stock solutions were prepared for each experiment by dissolving 3 mg CC (8.14 µmol) in 1 mL DMSO via short sonication, resulting in golden brown solutions. Similar dilutions of the curcumin stock solutions as used for the CD-CC loadings (see below) were used to prepare CC-NPs. For instance, 100 µL of the CC stock solution was added to 900 µL water and shortly vortexed. This solution was further diluted for UV–Vis measurements and cell experiments as described in the text.

4. Curcumin Loading of CD-NP:

To obtain different molar ratios of CD nanoparticles:curcumin (CD:CC), we added 50, 100, 200, or 300 µL of the above curcumin stock solution (0.407 to 2.44 µmol) to 700 to 950 µL of an aqueous solution containing 1 mg CD-NP each (ca. 0.8 µmol if assuming CD:TFTN = 1:1) contained in Eppendorf micro-centrifuge tubes, resulting in offered estimated molar ratios of 1:0.5, 1:1, 1:2, or 1:3 CD:CC. In cell experiments, the dosage was based on the weight:weight ratios of curcumin in the CD-NP host. Samples were briefly vortexed, placed in a thermal shaker for 15 minutes (37 °C, 660 rpm), and subsequently centrifuged at 1400 rpm (16873 rcf) for 10 minutes. Supernatants containing residual non-absorbed curcumin were collected and the curcumin content was determined by UV–Vis spectroscopy. Samples were refilled with 1 mL water and the washing process was repeated as above. A second washing resulted in clear supernatants. Concentrations of curcumin in the supernatants were determined with a calibration curve prepared from identical curcumin stock solutions, diluted in water, and read off at 430 nm. The combined concentrations were used to determine the total curcumin amount absorbed in the CD-NP host. As a combined result of the equilibration of curcumin with the CD-NP host and the washing step, typically 90% of the initially offered curcumin was absorbed in the CD-NP host. This lower real concentration was considered in the calculation of the effective cell exposure to curcumin (effective molarity values for determination of IC_{50}). The originally offered curcumin concentrations are displayed in all graphs.

5. Stability Studies of Curcumin:

5.1. Sample Preparation for UV–Vis Measurements:

CC-NP: 100 µL of a freshly made standard curcumin stock solution (3 mg/mL DMSO) was filled with water to 1 mL. Five samples were prepared by filling between 10 and 50 µL of this solution with water to 1 mL each, resulting in solutions containing between 3 to 15 µg CC/mL. Samples were repeatedly measured with UV-Vis spectroscopy over different time intervals.

CD-CC-NP: 1 mg CD-NP/900 µL was exposed to 100 µL CC/DMSO stock solution and treated as described under “loading of CD-NP”, resulting in a 1 mg/mL solution. Five samples were prepared by filling from 10 to 50 µL of this solution with water to each 1 mL each, resulting in solutions containing between 3 to 15 µg CC/mL. Samples were repeatedly measured with UV -Vis spectroscopy over different time intervals.

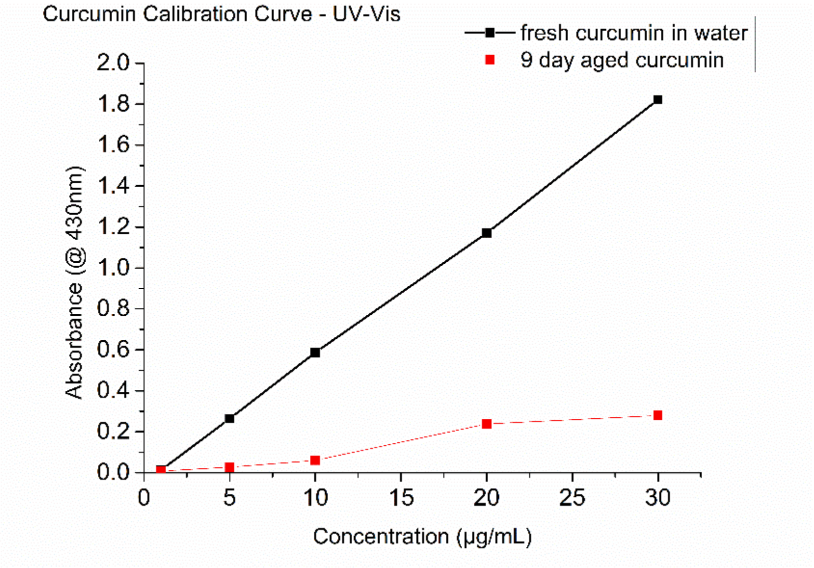

Figure 1. UV-Vis calibration curve of DMSO-dissolved curcumin in water; red curve: same solutions measured after 9 days when stored in the dark.

6. UV–Vis Spectroscopy

(Perkin Elmer Lambda1050 instrument with an InGaAs integrating sphere detector, Perkin Elmer, Hamburg, Germany). Aqueous sample solutions were measured in transmission in PMMA UV cuvettes with 10 mm path length, referenced against water.

7. Fluorescence Spectroscopy

(PTI spectrofluorometer with a photomultiplier detection system (model 810/814), Photon Technology International, Edison, NJ, USA . Excitation and emission slits were set at 2 mm, and an excitation wavelength of 420 nm was used. Measurements were performed in disposable UV PMMA cuvettes (Brand, Merck, Darmstadt, Germany), samples were stirred and kept at 37 °C.

8. Scanning Electron Microscope SEM

(FEI Helios Nanolab G3 UC, Eindhoven, Netherlands): The microscope was equipped with a field emission gun and was operated at acceleration voltages at 2–3 kV using working distances of about 3 mm. Samples were either prepared from aqueous solutions or were freeze dried and subsequently dispersed in ethanol or DMSO solution, shortly treated by ultrasonication and dropped on a Si wafer. Samples were dried over night before analysis.

9. Sorption Measurements

(Quantachrome NOVA 4000e, (now) Anton Paar, Graz, Austria). Nitrogen sorption isotherms were measured at 77 K. An amount of 10–15 mg of a freeze-dried sample was degassed for 12 h at 120 °C under vacuum (10^{-5} Torr) before measurement. Brunauer Emmett Teller (BET) surface areas were calculated using the linearized form of the BET equation (in the range of 0.08 to 0.2 P/P_0).

10. Dynamic Light Scattering (DLS) and Zeta Potential

(Malvern Zetasizer-Nano, 4 mW He-Ne laser (633 nm), equipped with a Multi-Purpose Titrator MPT-2, Malvern Panalytical, Nürnberg, Germany). The hydrodynamic particle size distribution was measured in aqueous solutions containing a concentration of 0.1 mg/mL CD-NP with three scans/sample. Zeta-potential measurements were performed with 10 mL of an aqueous solution of nanoparticles (0.1 mg/mL) using an automated titrator scanning the pH range from 2 to 8 (with pH steps of 0.5) using diluted NaOH and HCl solutions.

11. Thermogravimetric Analysis TGA

(Netzsch STA 440 C TG/DSC, Selb, Germany) Amounts of 5–10 mg of samples were heated with a heating rate of 10 K/min in a stream of synthetic air (with a flow rate of about 25 mL/ min).

12. Infrared Spectroscopy (FTIR)

Thermo Scientific Nicolet iN10 IR 6700 Microscope, ThermoFisher, Germany, in reflection–absorption mode with a liquid nitrogen-cooled MCT-A detector.

13. Raman Spectroscopy (FT-Raman)

(Bruker Equinox 55, equipped with the Raman Module FRA 106/S using an Nd/YAG laser at 1064 nm and a LN_2 cooled Ge detector, Bruker, Rosenheim, Germany) Spectra were collected in backscattering mode on powders. Usual set-up: accumulation of 5000 scans for CD samples, 1000 scans for TFTN and CC-containing samples with 100 mW laser power.

14. Cell Culture:

HeLa, human MD-MB-231 and MCF10A cell lines were obtained from ATCC, Wesel, Germany, T24 cells were obtained from DSMZ, Braunschweig, Germany. All cells were cultured in Dulbecco's modified Eagle's medium (DMEM), supplemented with 10% FBS, 100 U/mL penicillin and 100 µg/mL streptomycin at 37 °C and a 5% CO_2 humidified atmosphere. For cell experiments, cells were seeded in 96 well plates (Ibidi, 15 µ (bottom thickness) black, ibiTreat) and were allowed to adhere for 24 h before incubation with the respective NP in a Heracell Incubator (Thermo Fisher) at 37 °C and 5% CO_2.

15. Live-cell Imaging:

(ImageXpress Micro XLS, widefield high-content microscope, Molecular Devices, Wokingham, Berkshire, UK)) HeLa, MDA-MB 231 and T24 cells were each seeded with 5000 cells/well, MCF-10A cells with a density of 10,000 or 20,000/well in 100 µL DMEM. Curcumin nanoparticles (CC) or curcumin absorbed in CD-NP (CD-CC-NP) diluted to the required concentrations were added as 10 µl aliquot aqueous solutions to each well, were briefly shaken and immediately transferred into the life-cell imaging environmental control chamber of the high-content-screening wide-field microscope, and were kept at 37 °C under flow of a 5% CO_2 humidified atmosphere over the whole measuring time. Cells were incubated with curcumin for 1 to 3 h (termed "incubation time"), shortly removed from the environmental control chamber to perform a medium exchange, adding 100 µL medium without curcumin and replaced into the microscope chamber. The cell status was the further microscopically monitored for up to 3 days; this time span is referred to as "post-incubation time. Sequential images of the assigned wells in the plate were taken with 4 images for each well, automated scans were performed every 5 h over total periods ranging between 24 to up to 72 h. Objectives from Nikon were used with magnifications as indicated: 10× (Plan Fluor 0.3 NA), 20× (Ph1 S Plan Fluor 0.45 NA) or 40× (Plan Apo Lambda 0.95 NA). Bright field images were recorded in addition to fluorescence images (with GFP, DAPI, or CY5 dyes) as indicated using a filter cube changer (Semrock filters). Each experiment with the different cell lines was conducted between 3 to 6 times.

16. Image Analysis

Cell proliferation and live-dead assays were acquired from bright field images (usually at 20× magnification) of unlabeled cells. All samples were prepared in triplicates for each concentration in each experiment. In order to get statistically relevant results, we evaluated between 3 to 6 representative images from 3 different wells by hand-counting live and dead cells at different time points (between 50–700 cells/sample/time-point, depending on time-point in the experiment and growth status of the respective cells).

17. Viability Assays:

Endpoint analysis was additionally performed with cell counting Kit-8 assays (CCK-8, Dojindo, Munich, Germany) using the water-soluble tetrazolium salt which is reduced by dehydrogenases in living cells to a water-soluble orange formazan dye. 8 µL of this CCK-8 solution were added to each 100 µL well volume and were incubated for 2 h at 37 °C under 5% CO_2 atmosphere. The concentration of living cells was evaluated with a microplate reader (SpectraFluorTM Plus microplate reader S4, Tecan, Groeding, Austria) using absorbance at 450 nm by referencing against untreated cells in the same plate holder.

18. CD-NP characterization: Thermogravimetric analysis

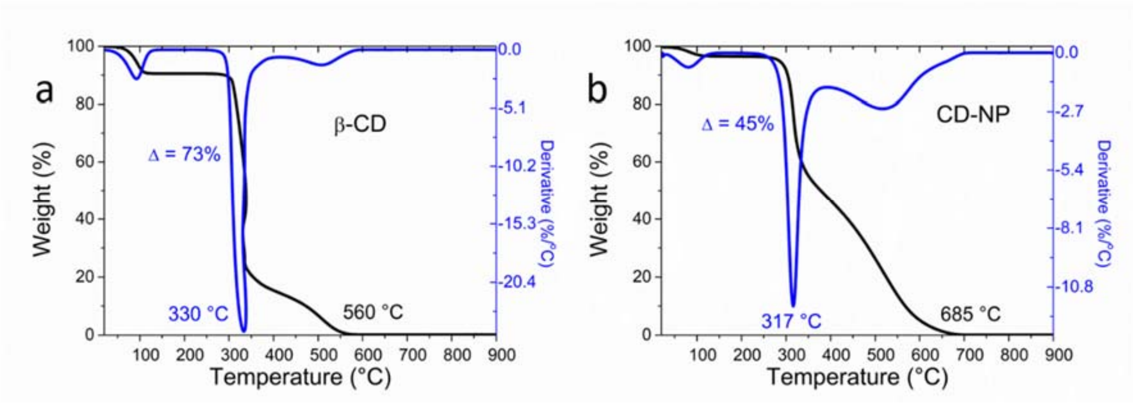

Figure S2. TGA thermogravimetric measurements of the precursor β-CD (a) results in a spontaneous decomposition at 330 °C in contrast to a gradual structural break-down of the TFTN crosslinked CD-NP (b).

19. CD-NP Characterization: Nitrogen Sorption

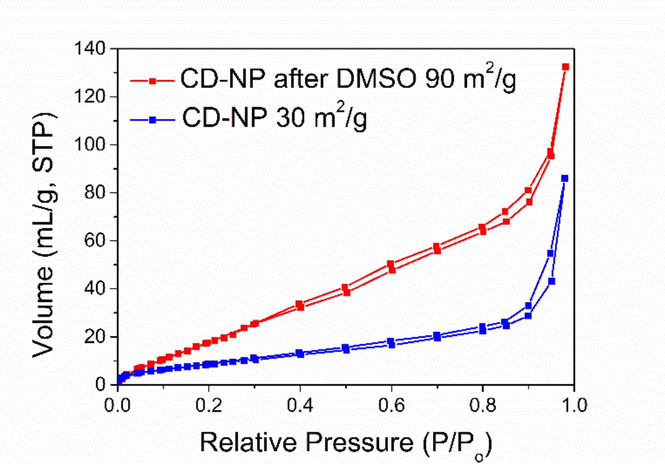

Figure S3. Nitrogen sorption isotherms of crosslinked CD-NP blue: freeze-dried sample retrieved from aqueous suspensions red: freeze-dried samples retrieved after an additional DMSO wash.

20. Loading Efficiencies of Curcumin in CD-NP

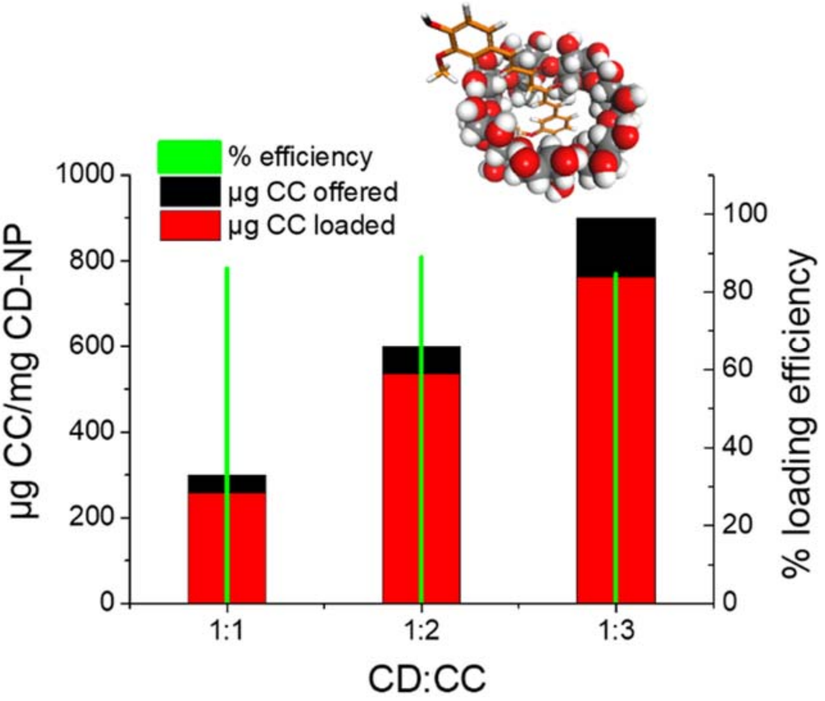

Figure S4. Loading efficiencies of curcumin in crosslinked CD-NP. Aliquots of 1 mg CD-NP in 1 mL water were exposed to 300, 600 or 900 µg curcumin (100, 200 or 300 µL of a curcumin stock solution of 3 mg CC/mL DMSO) representing a molar ratio of CD:CC of 1:1, 1:2 and 1:3. Samples were washed 2× and the CC content in the supernatant was determined by UV-Vis spectroscopy. The optimized assembly of curcumin in one β-CD cavity as calculated with the Materials Studio package is shown on top.

21. Particle Size Stability of DMSO-Derived Pure Curcumin Nanoparticles

Aqueous CC-NP solutions were prepared from a DMSO stock solution and the hydrodynamic particle size was measured with DLS. We found that the particle size changed dramatically with concentration and time, indicating a significant aggregation/de-aggregation behavior.

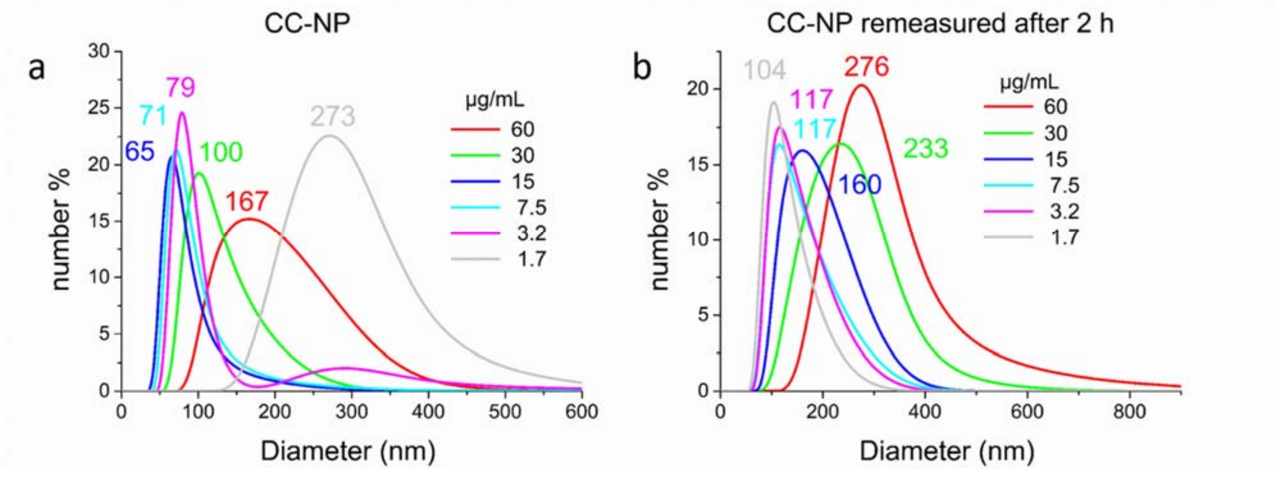

Figure S5. DLS-derived particle size distributions of curcumin particles in aqueous solution (**a**) directly after preparation and (**b**) after standing in the dark for 2 h. The PDI of freshly prepared DMSO-derived curcumin particles were in the range between 0.06 and 0.19. Particle size is strongly dependent upon CC concentration and time.

22. Photostability of DMSO-Derived Curcumin Nanoparticles in Comparison to Curcumin Encapsulated in CD-NP: UV–VIS Spectroscopy

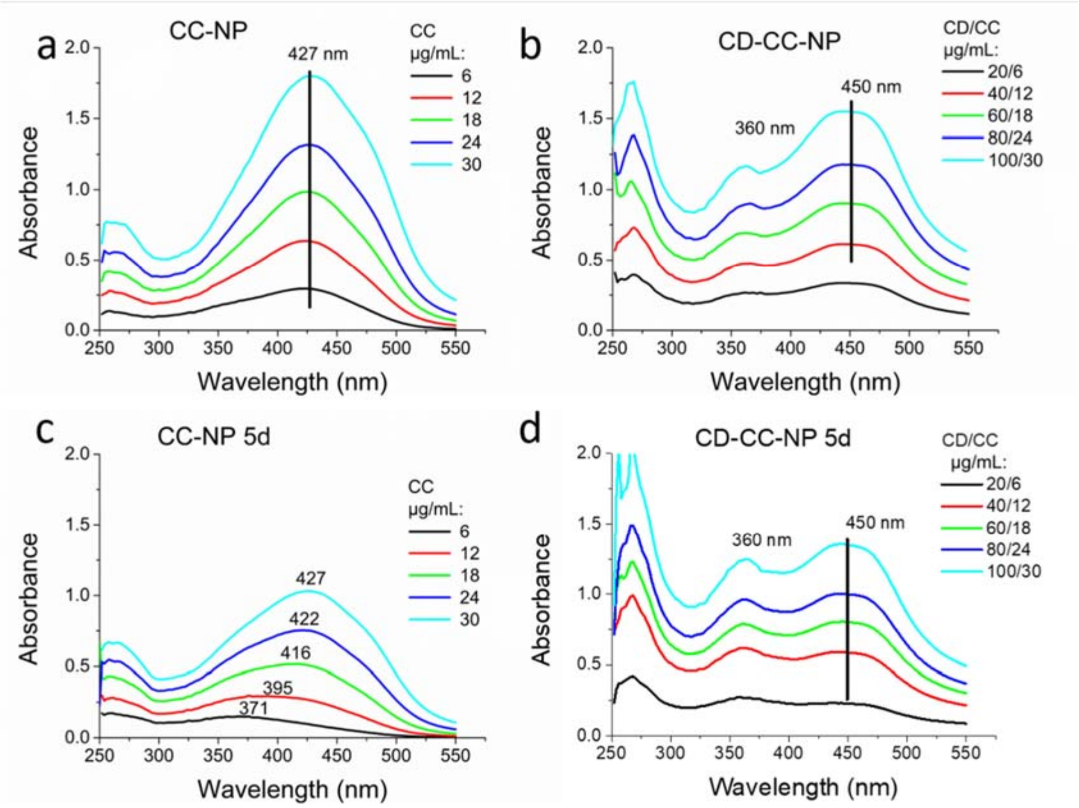

Figure S6. UV-Vis spectra of aqueous solutions of freshly made curcumin nanoparticles (CC-NP) with increasing concentrations and equivalent concentrations of curcumin absorbed in CD-NP (sample CD-CC-NP). (**a,b**) Spectra were measured directly after preparation, (**c,d**) after being exposed to daylight for 5 days. The respective concentrations are indicated in the figures.

Figure S6 shows the UV–Vis spectra with increasing curcumin concentrations taken immediately after sample preparation and after standing in air at room temperature for 5 days. The fresh CC-NP solutions show the typical absorption spectrum of curcumin with an absorption maximum at 427 nm, linearly increasing with increasing concentrations. Theoretical DFT calculations relate this peak to the enol form of curcumin [1]. Similar concentrations of the CD-CC-NP showed a red shift of the absorption maximum to 450 nm, once again indicating an intimate interaction of curcumin with its host. Additional features in the UV-Vis spectrum at 360 nm and 267 nm originate from the CD-NP carrier. When these solutions were remeasured after standing in ambient light for five days, we noticed drastic changes in the CC-NP samples. The absorbance was nearly halved and the absorption maxima were shifted towards shorter wavelengths as a function of the CC-NP concentration, indicating a concentration-dependent hydrolytic degradation [2]. In contrast, the CD-CC-NP spectra were nearly unaffected (Figure S6c,d).

A similar strong decay of UV-Vis absorption was observed for the CC-NP even when the samples were kept in the dark (see Figures S7 and S8). A minor decay in absorption is recognized during the first few hours even for the CD-CC-NP samples, but these values leveled out close to the original intensities. Conversely, CC-NP samples showed a rapid decay at the start and a continuing decrease to very low absorption values.

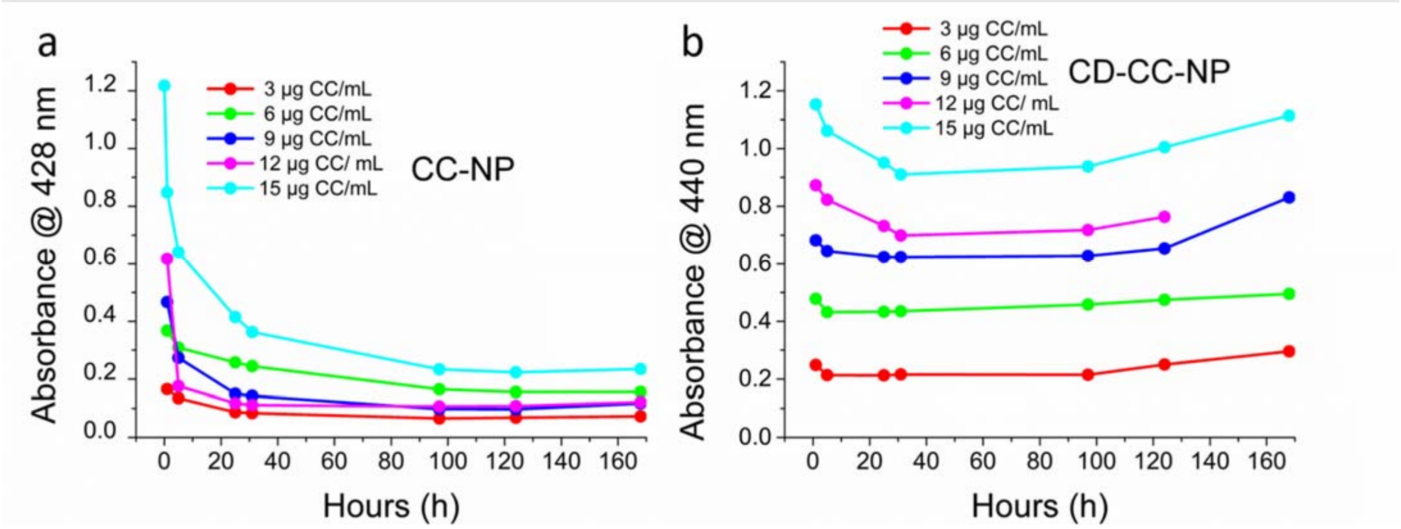

Figure S7. UV-Vis long term stability measurements over 7 days of (**a**) CC-NP and (**b**) CD-CC-NP in aqueous solutions containing comparable curcumin concentrations. Samples were stored in the dark between measurements. Absorption values were taken at the spectral maximum at either 428 nm for CC-NP (slight shifts were observed after 4 days) or at 440 nm for CD-CC-NP.

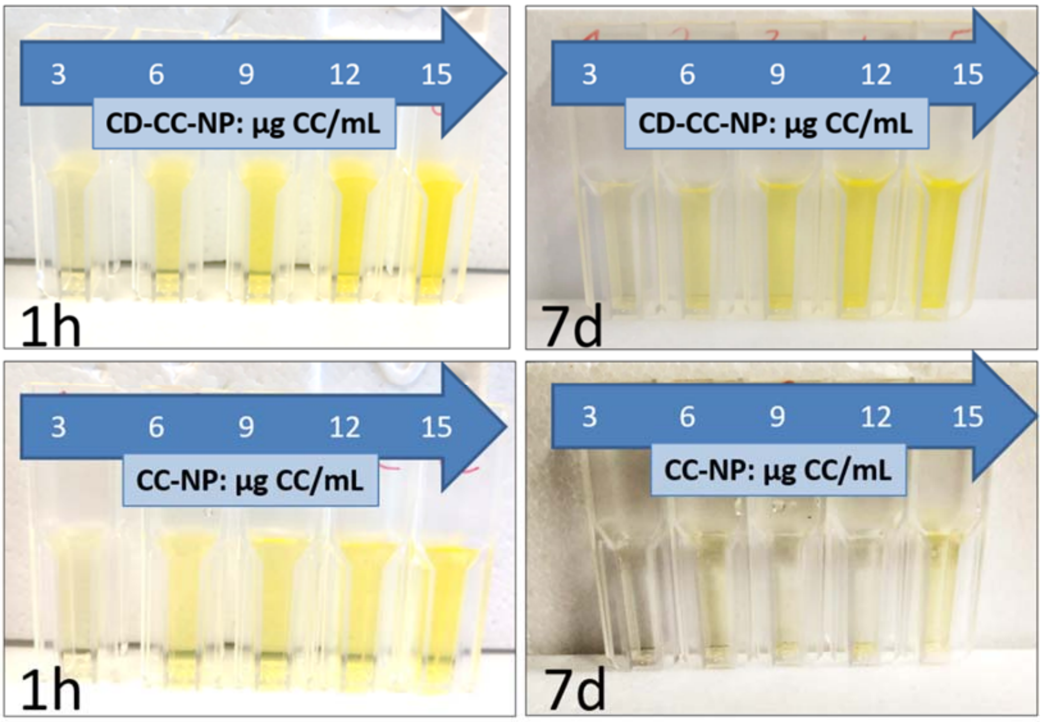

Figure S8. Photographs of CC-NP and CD-CC-NP samples as presented in Figure S7, taken at the start and the end of the measurements, showing the bleaching of the free curcumin samples CC-NP (bottom).

23. Medium-Dependent Release of Curcumin from CD-NP

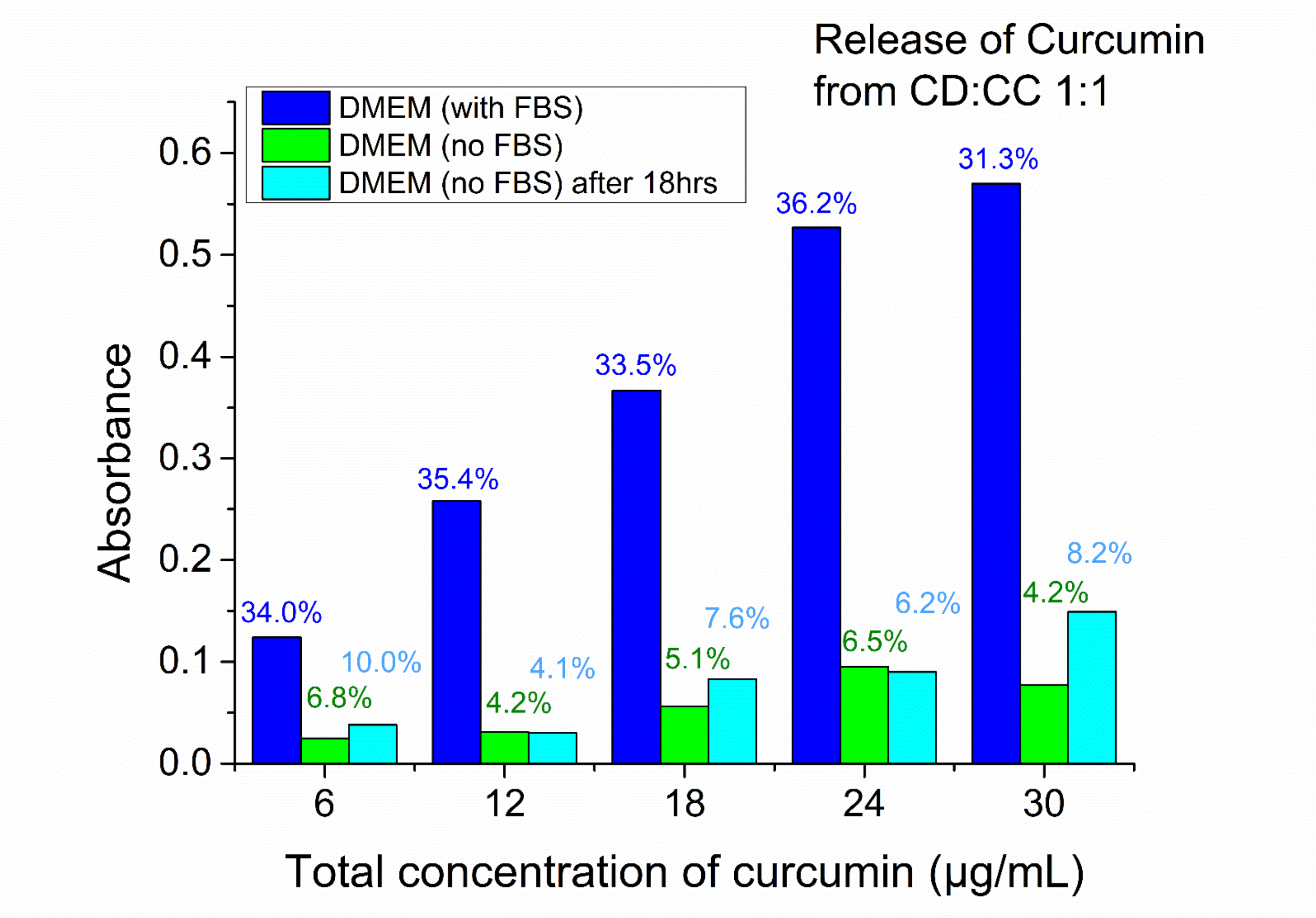

Figure S9. UV-Vis measurements of released curcumin from CD-CC-NP in cell culture medium. Increasing amounts of a CD-CC sample loaded with curcumin at a molar ratio of 1:1 CD:CC were suspended in either fluobright DMEM with or without additional 10% FBS, were shortly vortexed and shaken for 15 min at 37 °C before being centrifuged at 1400 rpm for 10 min. The supernatants were subsequently measured by UV–Vis as well as fluorescence spectroscopy (see below, Figure S10). The maximum absorption values at 425 nm are displayed. When exposed to DMEM (either red or fluobrite) only small amounts of curcumin were found in the supernatants (green bars). The concentration increased only slightly when the samples were shaken for 18 h in this medium (light blue bars). No measurable release was found when DPBS or water was used. However, when DMEM was enriched with 10% FBS, as used in cell experiments, the released curcumin concentration increased to over 30% of the absorbed amount in the respective CD-CC-NP.

24. Fluorescence Spectroscopy of Curcumin

When DMSO-derived curcumin nanoparticles were dispersed in water at different concentrations, we observed an emission spectrum at about 550 nm (excitation at 420 nm). In contrast to the UV–Vis spectra, we found a nonlinear relationship of curcumin emission intensity with concentration, indicating quenching, Figure S10a. Studying the potential desorption of curcumin from CD-CC nanoparticles, we did not see any release as long as these particles were immersed in water, DMEM or DPBS, Figure S10b. However, similar to our observation with UV–Vis spectroscopy, a concentration-dependent elution of curcumin from the CD nanoparticles was measurable as soon as 10% FBS was present in the medium. Association of the released curcumin with the proteins is indicated by an emission shift down to about 490 nm.

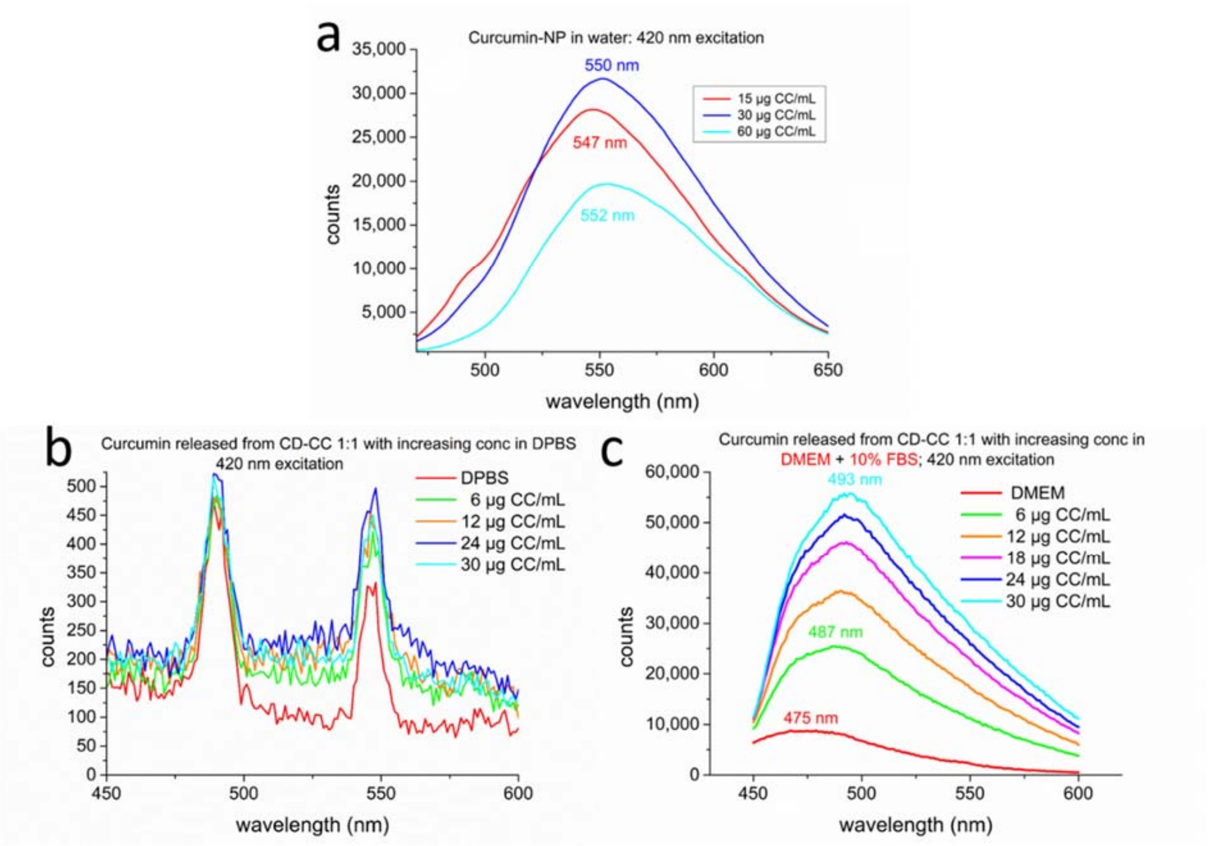

Figure S10. Emission spectra of curcumin; (**a**) emission of pure DMSO-derived curcumin particles diluted in water, (**b**) no emission was visible when CD-CC-NP with different concentrations were suspended in DPBS. Only extremely weak emission signals from the cuvettes are recognized. (**c**) Curcumin was eluted from CD-CC-NP when samples were exposed to DMEM containing 10% FBS.

25. Apoptotic Response of HeLa Cells after Pure Curcumin Nanoparticle Delivery

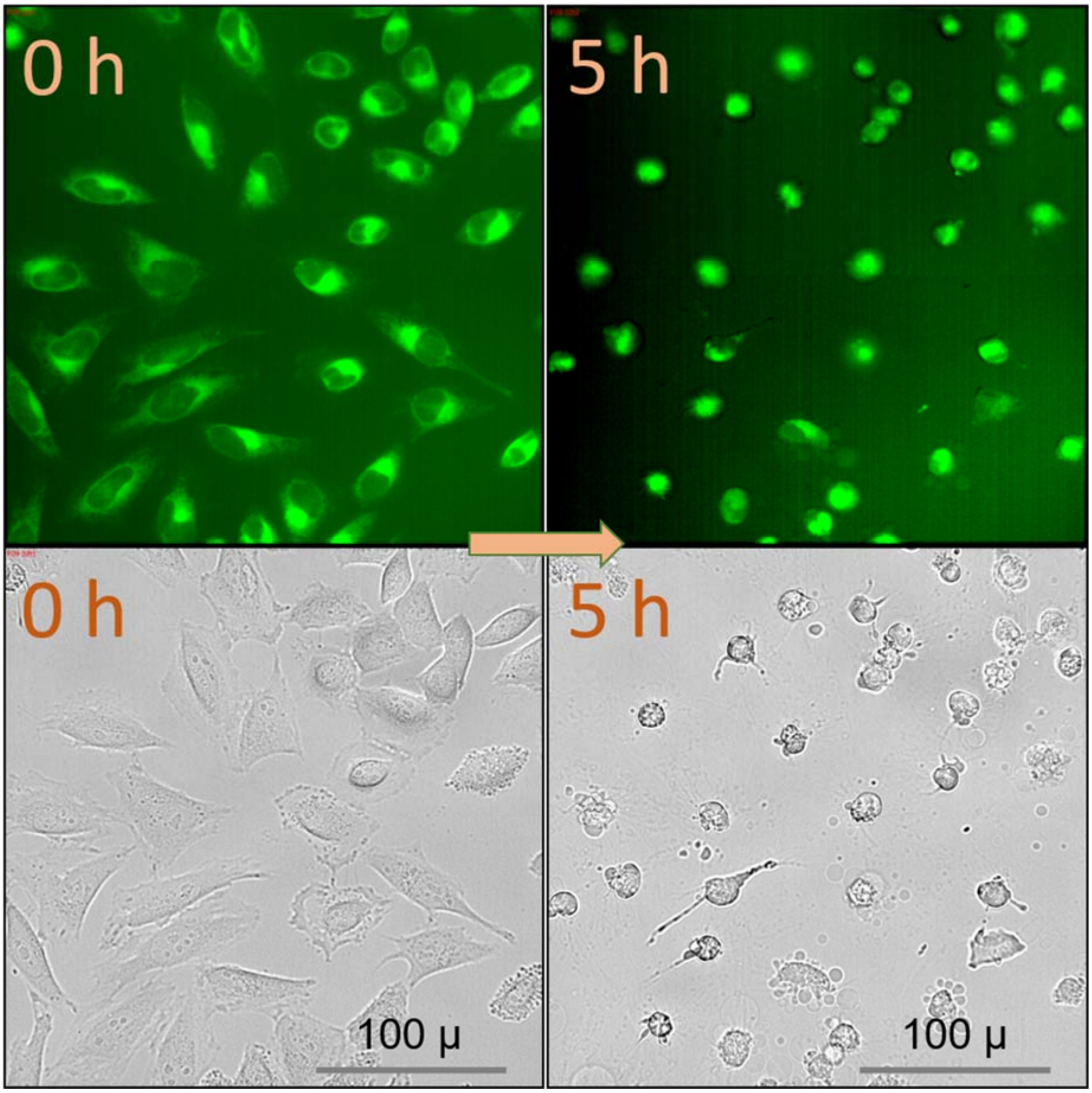

Figure S11. Fast apoptotic response was observed upon curcumin exposure in form of pure curcumin nanoparticles, similar to the curcumin delivery with CD-CC-NP (see Figure 4, main text). Images of HeLa cells treated with DMSO-derived CC-NP: 1 h incubation, 0 h post-incubation (left) and 5 h post-incubation (right) with 3 μg CC-NP (per well; 81 μM).

26. Concentration-dependent Kinetic Response Curves upon Delivery of Pure Curcumin Nanoparticles to HeLa Cells

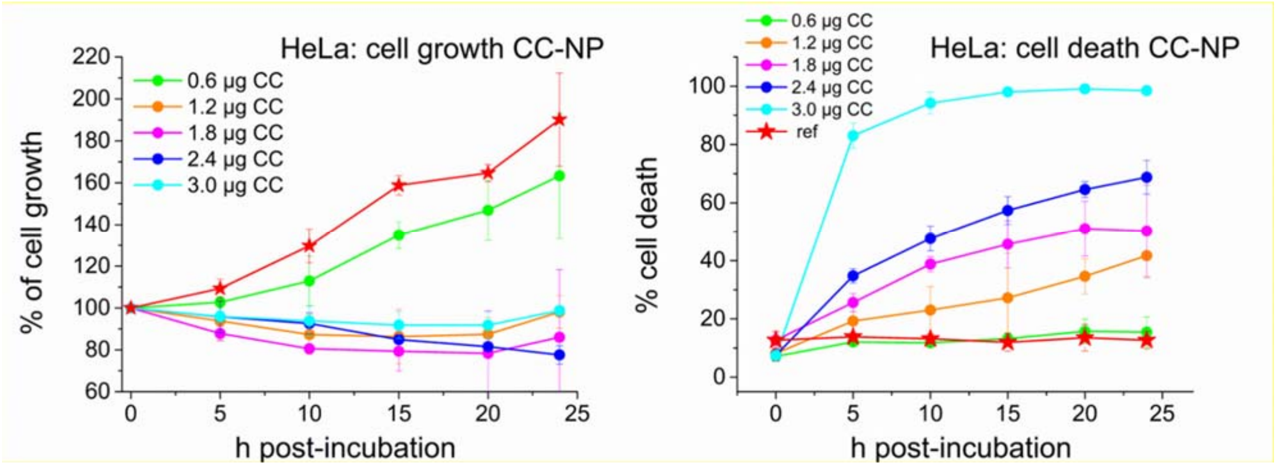

Figure S12. Concentration-dependent kinetic response curves displaying cell growth (left) and cell mortality (right) of HeLa cells following a 1 h incubation time with DMSO-derived pure CC-NP. The cell behavior was similar to the results obtained when curcumin was delivered with CD-CC-NP: cell growth was inhibited already at low CC concentrations. However, curcumin delivered in this formulation needs the highest concentration of 3 µg CC/100 µL for nearly complete apoptosis, substantially more than with CD-CC-NP (see Figure 5, main text). In all our experiments performed with HeLa cells, we noticed a slightly lower cytotoxicity of the pure CC-NP relative to CD-NP delivered curcumin.

27. Influence of Curcumin Loading by Changing the CD:CC Molar Ratio in HeLa Cells

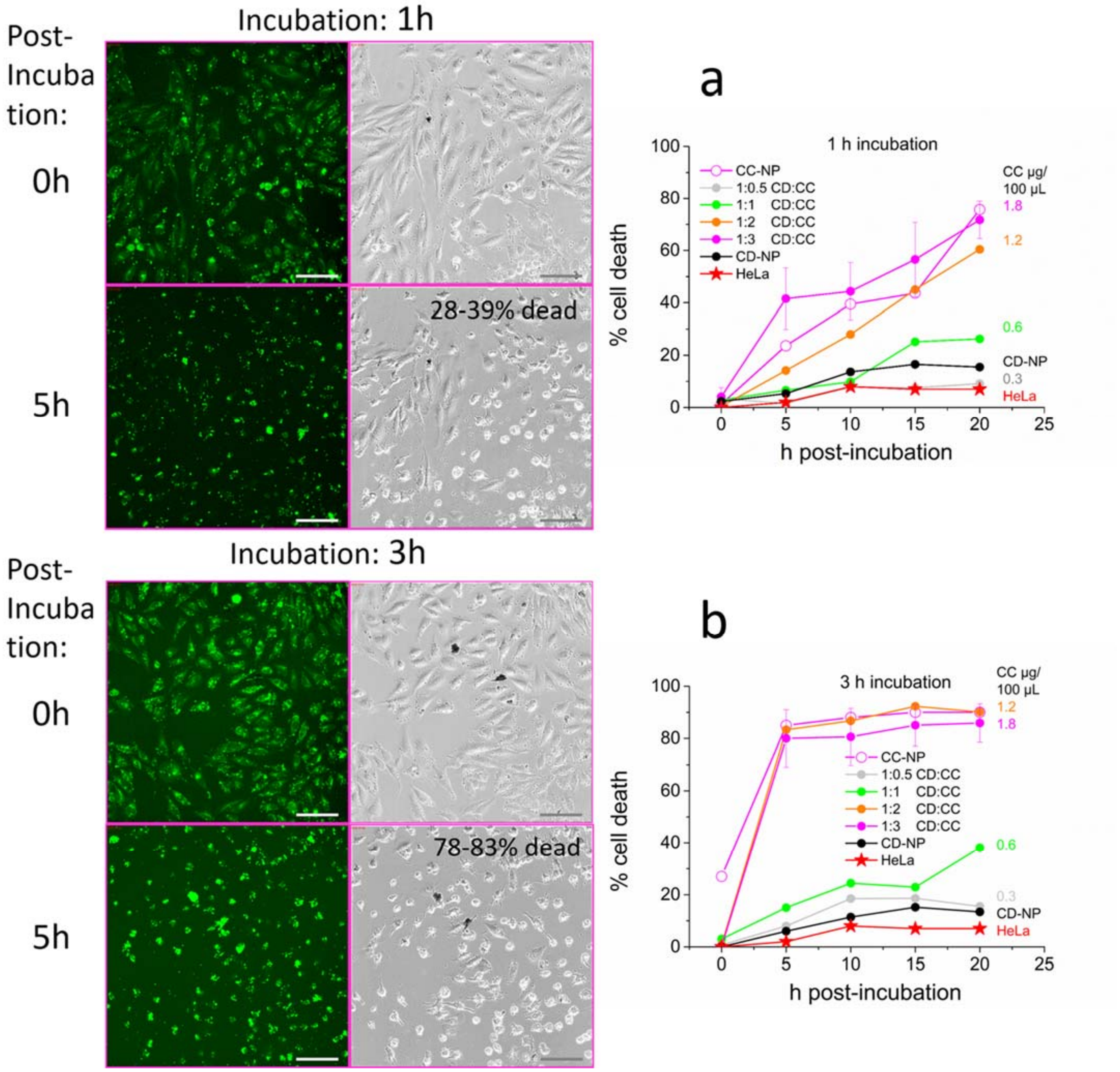

Figure S13. Increasing curcumin delivery by increasing the CC loading in CD-NP using (**a**), 1 h and (**b**), 3 h incubation time: cells are exposed to a constant CD-NP concentration of 2 µg/well in 100 µL. CD-CC-NP samples were prepared with increasing CD:CC molar ratios, ranging from 1:0.5 to 1:3; 2 µg CD-CC-NP correspond to 0.3 µg up to 1.8 µg CC/well. Representative images (1.8 µg CC/100 µL; CD:CC 1:3) are shown directly after medium change and after 5 h post-incubation time (incubation time in (**a**): 1 h, in (**b**): 3 h; scale bar: 100 µm). Dead cell concentrations at 5 h post-incubation are indicated in the respective images. Concentration-dependent kinetic response curves reflecting cell death development are shown on the right. Pure CD-NP (2 µg/well) and pure CC-NP (1.8 µg/well) are included. Comment to (**a**)**: 1 h** incubation results only in a cell mortality of about 40% after 5 h transfection as compared to 80% in Figure 5 main text with a CD:CC mol ratio of 1:1. However, after 20 hour post-incubation 65–79% cell death is achieved (1.8 µg CC/well).
Comment to (**b**)**: 3 h** incubation time increases the mortality rates: 80% mortality is achieved again after 5 h post-incubation. The IC_{50} value is now decreased to 32 µM (1.2 µg CC/well (100 µL)).

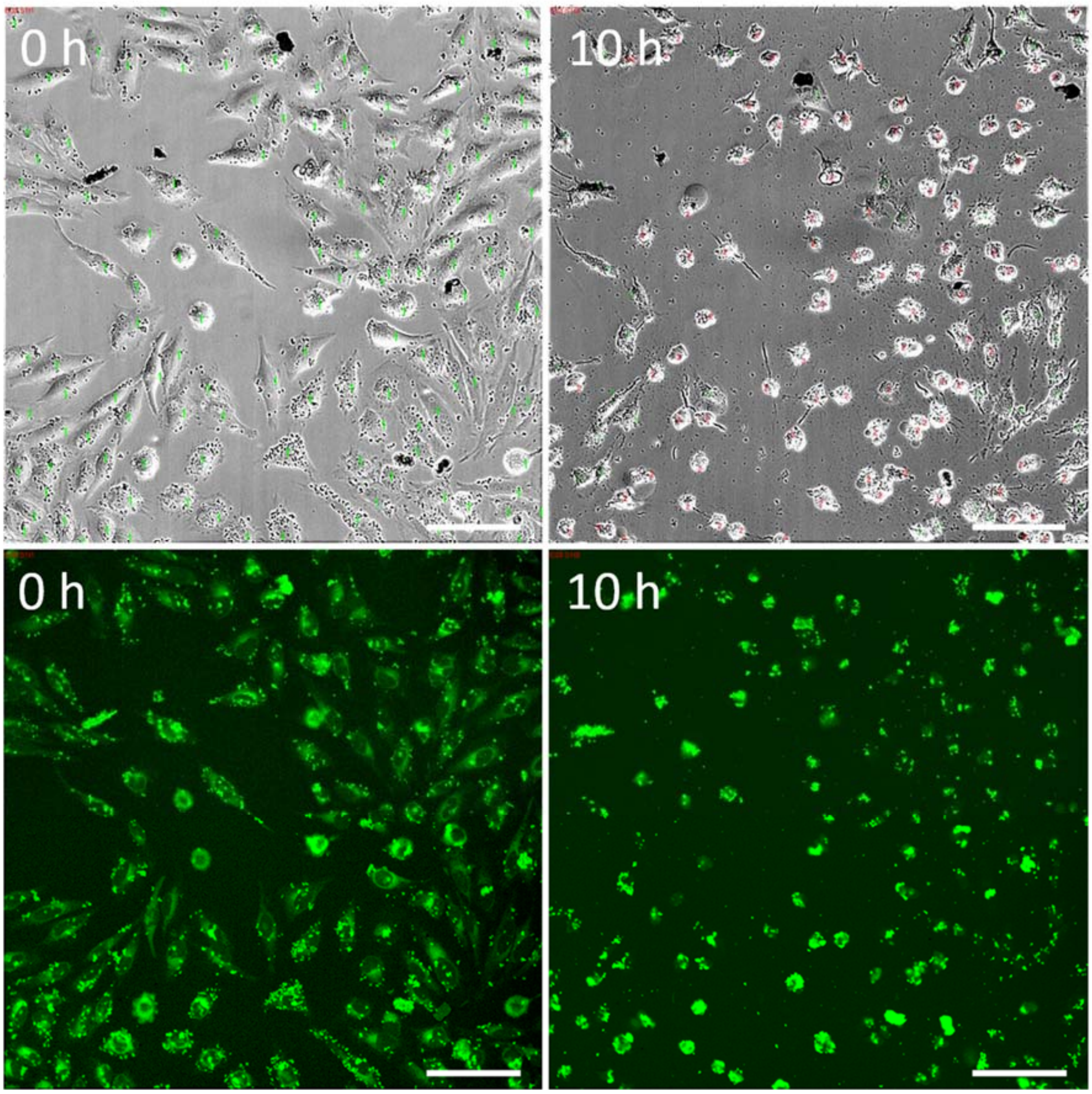

Figure S14. Example for cell counting: representative images after 3 h incubation and 0 h, 10 h post-incubation time with 1.8 µg/well CC in CD-CC-NP (CD:CC 1:3 molar ratio; scale bar: 100 µm). Green labeled cells were counted as alive, red labelled as dead (color labels in transmitted light micrographs are kept small for better cell visibility). Even visually unhealthy cells were counted as alive as long as no blebbing or minor microtubule spikes were recognized and cells still showed some spreading.

28. Impact of Curcumin Loading by Changing the CD:CC Molar Ratio: Endpoint Analysis (Cell Counting) HeLa Cells

HeLa cells were incubated for different incubation times (1, 2 and 3 h) with CD-NP carrying an increasing load of curcumin (molar ratios CD:CC of 1:1, 1:2 and 1:3) and were monitored over a 20 h post-incubation time. When an end-point analysis was made by counting the number of dead cells, we noticed that the incubation time had no major influence on the final result after 20 h (see Figure S15). Cell death was between 63 to 81 % when 1.8 µg curcumin/well (mole ratio 1:3) was delivered either as pure curcumin nanoparticles CC-NP or when loaded in CD-CC-NP. However, when analyzing the time trace (see Figure S13) we noticed that a short incubation time slowed down the cell killing noticeably. It took 20 h to achieve about 70% cell death with 1-hour incubation time, but with 3 h incubation time a similar result was already achieved within timeframes shorter than 5 hours.

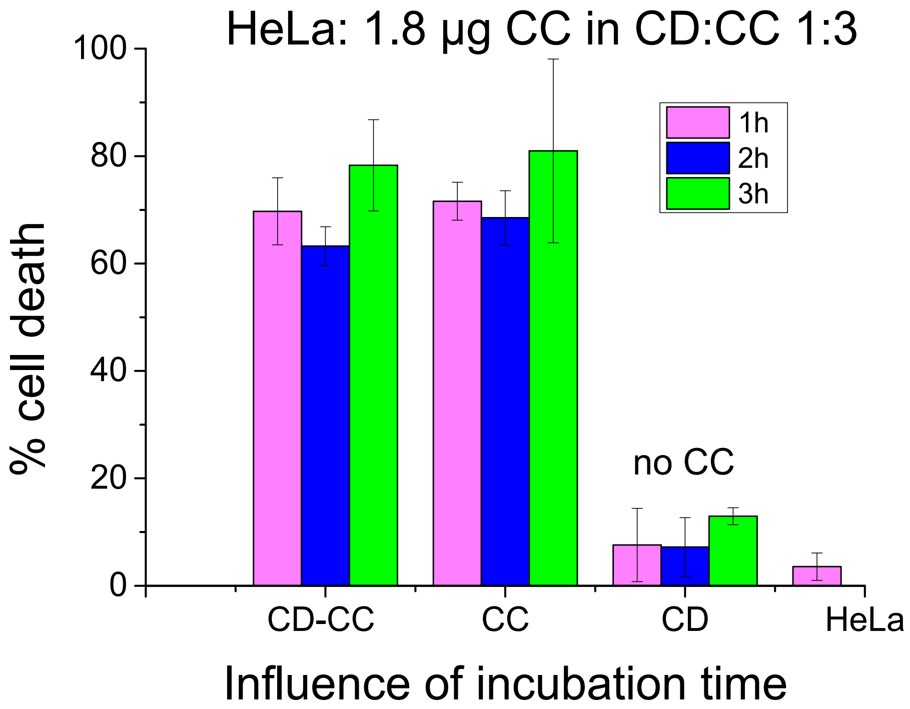

Figure S15. Change of incubation time: 1, 2 or 3 h. Endpoint analysis by cell counting. Cell mortality after 20-hour post-incubation time with 1.8 µg CC/well in CD-CC-NP (molar ratio of 1:3) or delivered as CC-NP. The mortality of cells treated with empty CD-NP carriers and of untreated HeLa cells is included for reference.

29. Development of Cytoplasmic Vacuolization in HeLa Cells

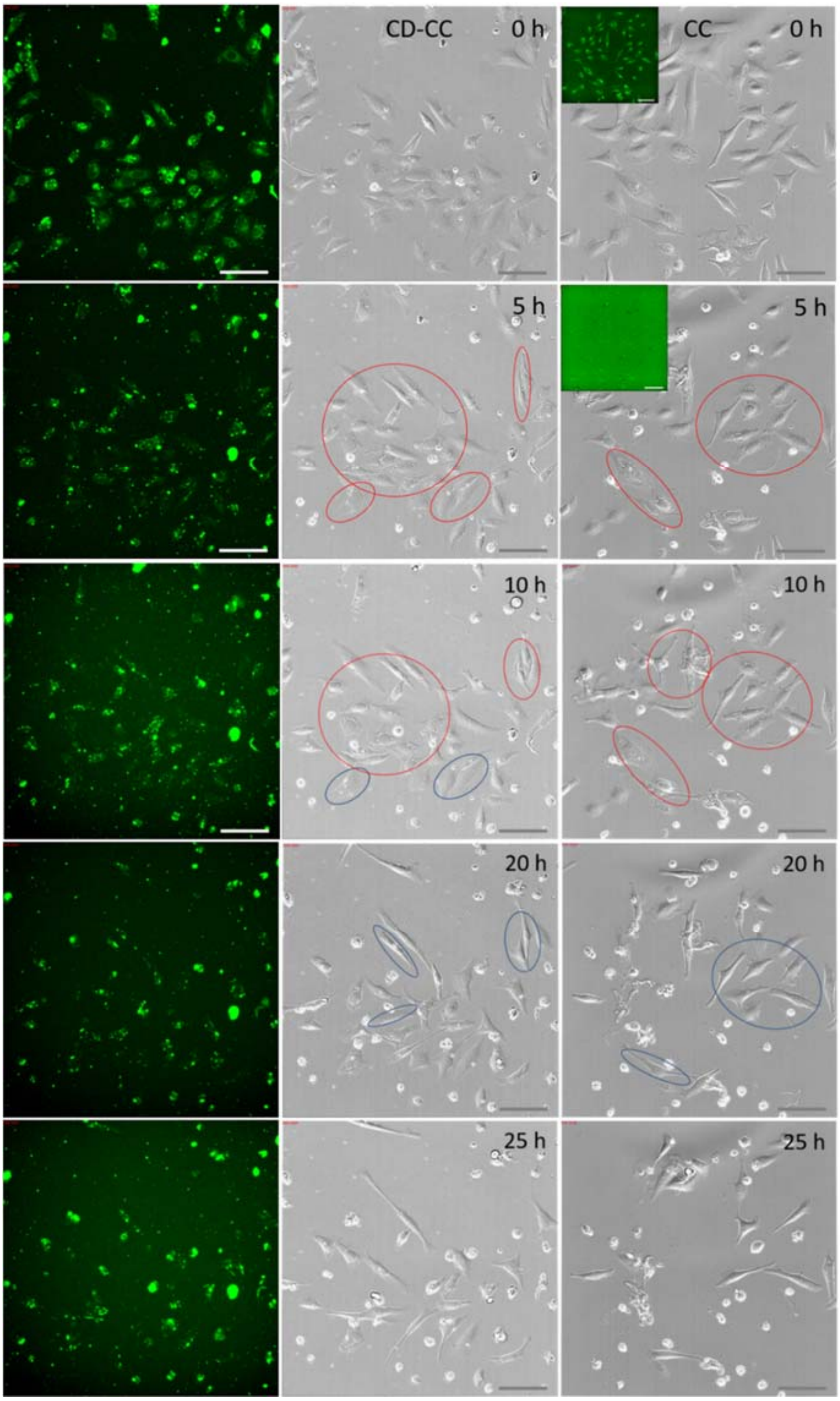

Figure S16. Micrographs of HeLa cells taken directly after medium change (after 1 h incubation) until 25 h post-incubation. Cells were treated with 1.8 µg CC/well either as CD-CC-NP (left, GFP channel and bright field images) or as CC-NP (right, bright field images; GFP channel images of these samples are shown as insets only at 0 h and after 5 h because fluorescence intensity had dramatically diminished after this time). Cells that have developed vacuoles are circled in red. Some cells that were able to recover into their original appearance are circled in blue. Scale bar = 100 µm.

30. HeLa Cell Labeling with Mitochondrial Tracker CMXRos

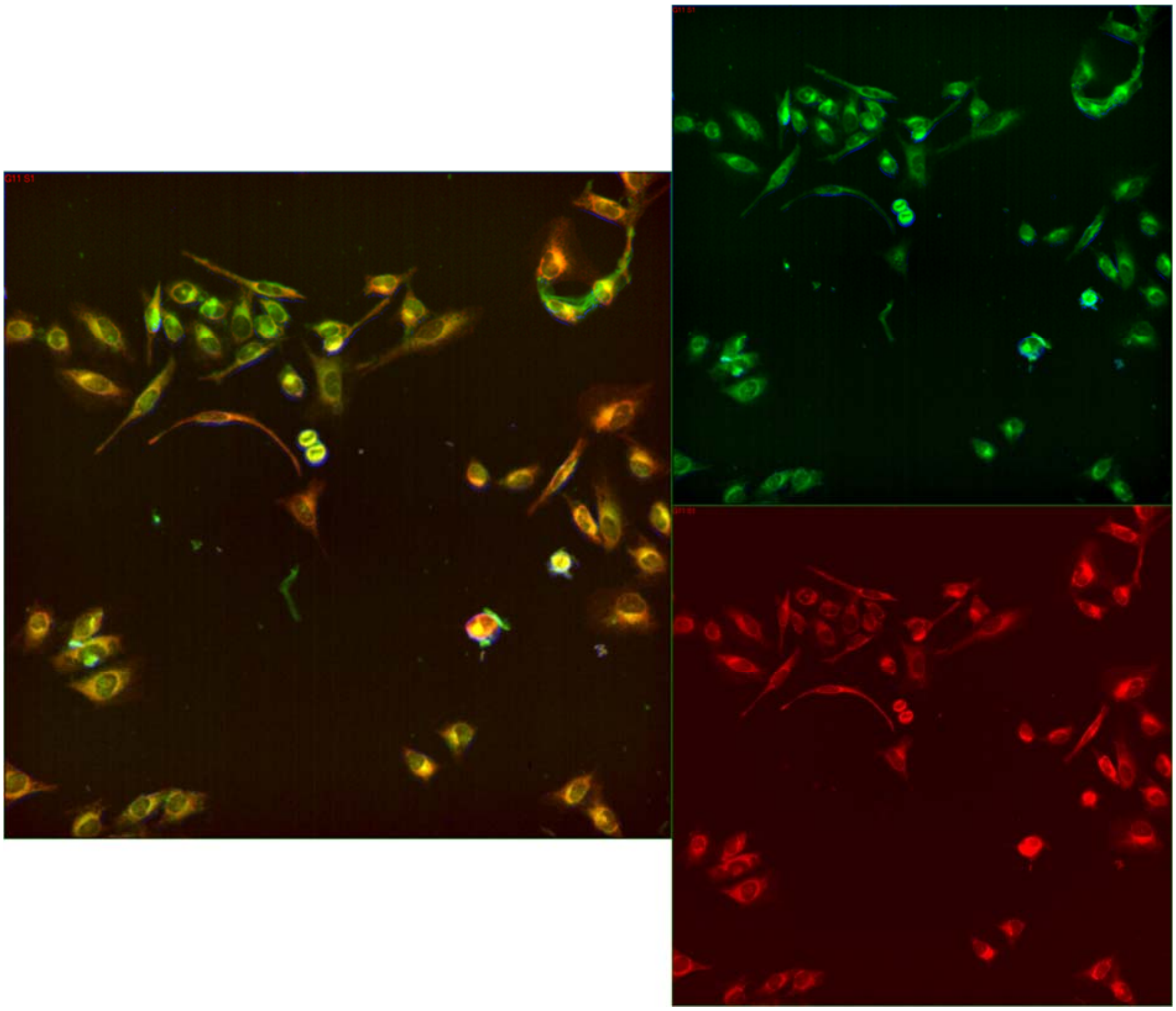

Figure S17. Micrographs of Hela cells incubated with 2.4 µg/well curcumin for 1 h and subsequently labeled with mitochondria-trackerRED CMXRos. The overlay (left) shows an overlap of curcumin and mitochondria only in some of the cells.

31. Cytotoxicity of Aged Curcumin Samples

In order to study if the photostability and antitumoral activity of curcumin is related, we performed equivalent experiments as described in Figures 4 and 5 main text, however, now using samples that were exposed to daylight for 5 days. The same concentrations were used, a 1 h incubation time was again applied and cells were observed over a period of 48 h after medium change. In Figure S18 we document only the results for the highest concentration applied (3 µg CC as pure CC-NP and absorbed in 10 µg CD-NP/well; CD:CC 1:1; for other concentrations see Figure S19). We display the total number of untreated HeLa cells at start, after 24 or 48 h post-incubation in green, while the total number of cells treated with CC-NP or CD-CC-NP is shown in red. The respective fraction of living cells is shown in grey. All results are normalized to the non-treated reference after 24 or 48 h. For comparison, we display the results obtained when all samples were prepared fresh, analyzed after 24 h in Figure S18a, while Figure S18b and c shows the results for aged samples after 24 h and 48 h, respectively. Comparing the fresh samples in Figure 18a and the aged samples in Figure 18b, it is obvious that the usually highly active curcumin nanoparticles CC-NP lose their anti-cancerous properties nearly completely when aged. The cell growth is nearly identical after aging, but 91% of the total cells are still alive in the aged sample, almost unchanged to the reference, where 93% of all cells are alive after 24 h. In contrast, curcumin delivered via CD-NP is only mildly affected by aging: mitosis is almost completely suppressed as the cell concentration has not changed much with respect to the starting conditions, and 52% of the existing cells have died. This situation is even more pronounced after 48 h post-incubation for CD-NP: cells did not grow during this time and slightly more

(60%) of the total cells are now dead, in stark contrast to the aged CC-NP samples. Representative images of CC-NP and CD-CC-NP treated cells and of the HeLa reference in Figure 18d reflect the influence on mitosis and cell killing after 48 h. Thus, encapsulation of curcumin does not only preserve the auto-fluorescence of curcumin but clearly also its anti-tumor properties.

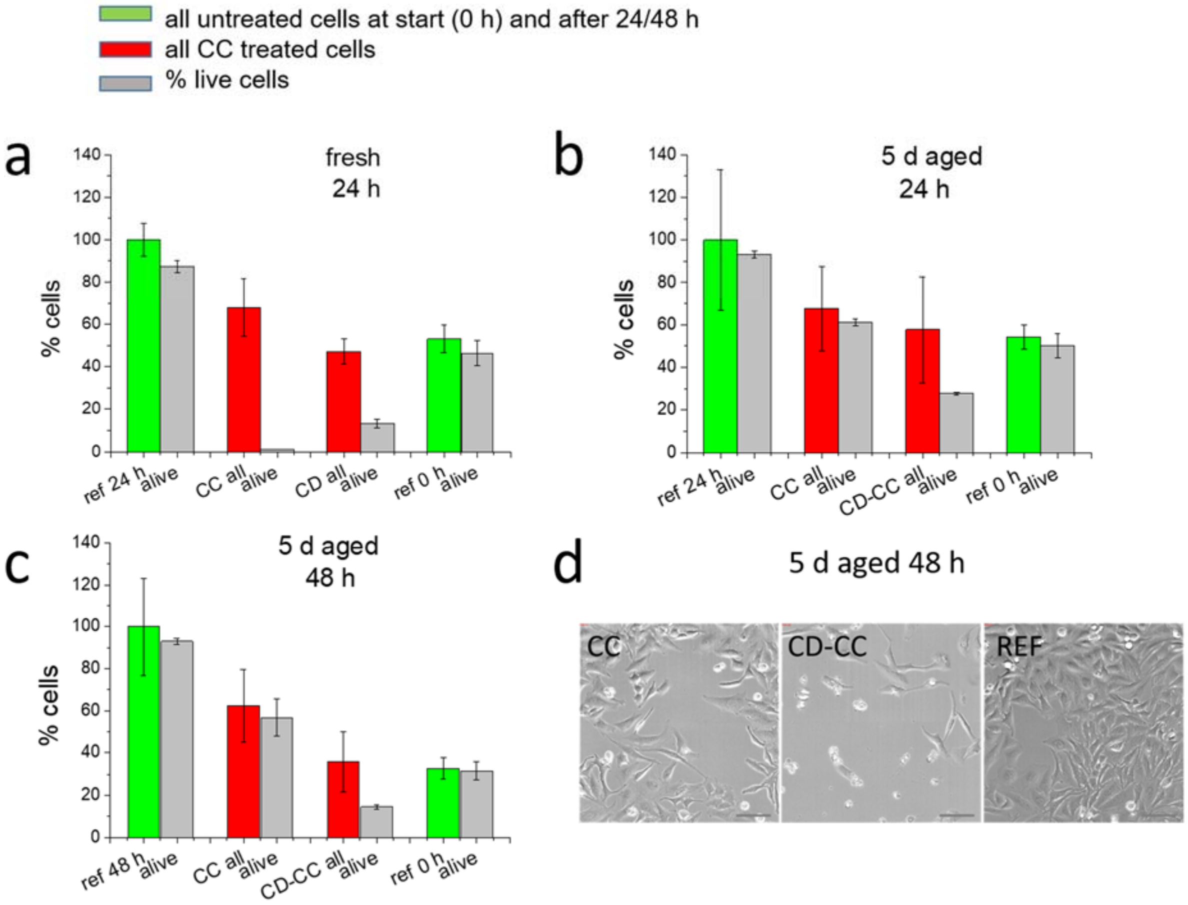

Figure S18. Endpoint analysis of HeLa cells by cell counting after treatment with CC and CD-CC NP delivering 3 μg of curcumin (1 h incubation): (**a**) cells treated with freshly made samples, (**b**) cells treated with 5d aged samples, where cells were analyzed after 24 h post-incubation, and (**c**) identical cells analyzed after 48 h post-incubation, (**d**) representative images after 48 h post-incubation with aged samples; scale bar = 100 μm. All cell counts were normalized to the untreated reference at 24 h or 48 h, respectively.

32. Concentration-Dependent Curcumin Activity on HeLa Cells Using 5 Days Aged Samples

Endpoint analysis after 48 h post-incubation with 5-day aged samples shows the superiority of curcumin samples enclosed in CD-NP in Figure S19: while aged CC-NP induce only a minor reduction of cell mitosis (overall concentration of cells normalized to untreated cells after 48 h (ref 48 h) is only reduced by maximal 40%) and nearly no cell-killing effect is observed, we do see a nearly complete cell cycle arrest and a larger cell-killing with higher CD-CC-NP concentrations even after aging.

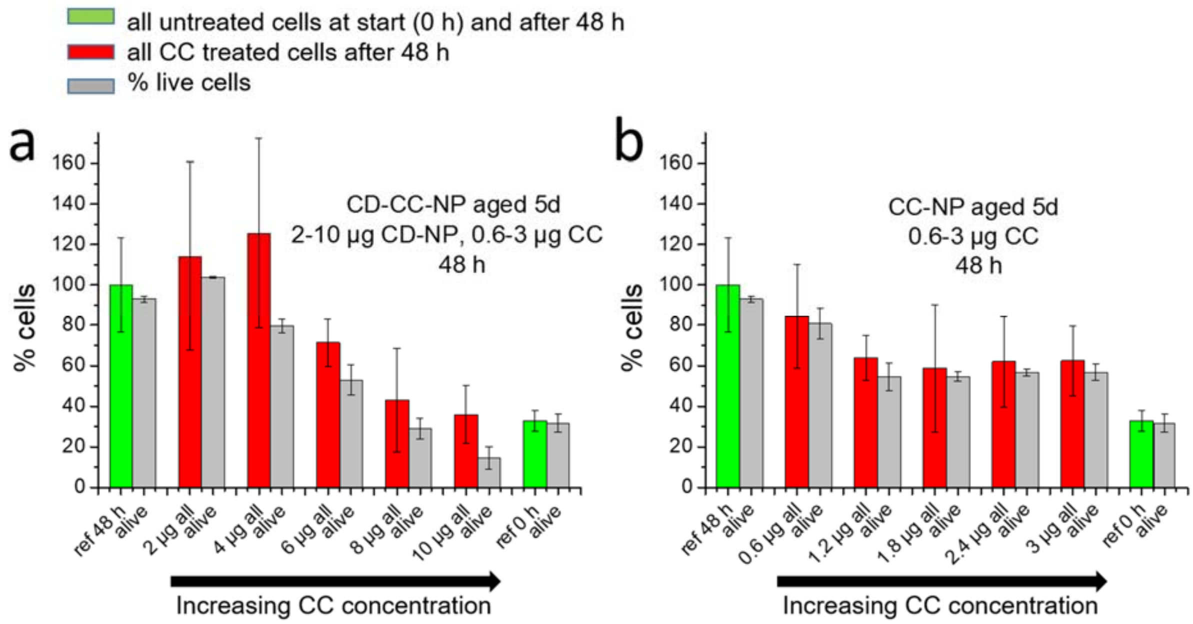

Figure S19. Impact of curcumin aging on HeLa cells. HeLa cells were incubated for 1 h with 5 day-aged samples (in daylight), with curcumin adsorbed in (**a**) CD-CC-NP or in (**b**) with equivalent concentrations as pure DMSO-derived CC-NP. An endpoint analysis was performed by cell counting after 48 h. Green: total number of untreated HeLa cells at start and after 48 h, red: total number of CC or CD-CC-NP treated HeLa cells, grey: living cells; all cell counts were normalized to the untreated reference at 48 h.

33. Effect of Curcumin on T24 Cells

Bladder cancer cells demanded a longer incubation time with curcumin. After 1 h incubation with 1.8 µg CC/well as either CC-NP or CD-CC NP resulted in nearly no dead cells (see Figure S20, red bars). However, after 3 h incubation time mortality increased dramatically when this amount was delivered as CC-NP. CD-CC-NP activity was comparable only at higher concentration as seen in the corresponding images in Figure S21, where 3 µg CC/well delivered *via* CD-CC-NP caused massive cell death again (see also Figure 9 in the main text). In addition, we noted increasing exocytosis of curcumin-containing agglomerates in time.

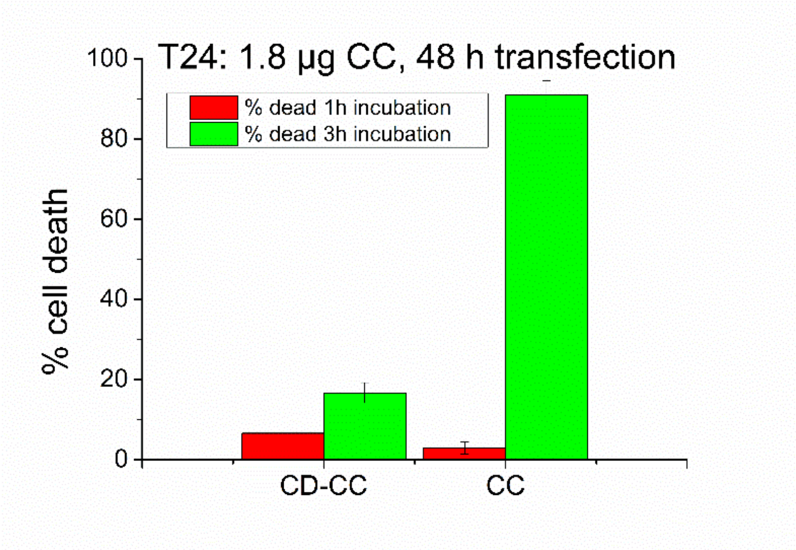

Figure S20. Cell mortality of T24 cells after 1 h (red) or 3 h (green) incubation time and 48 h post-incubation time. 1.8 µg CC/well was delivered either in CD-CC-NP or as pure CC-NP.

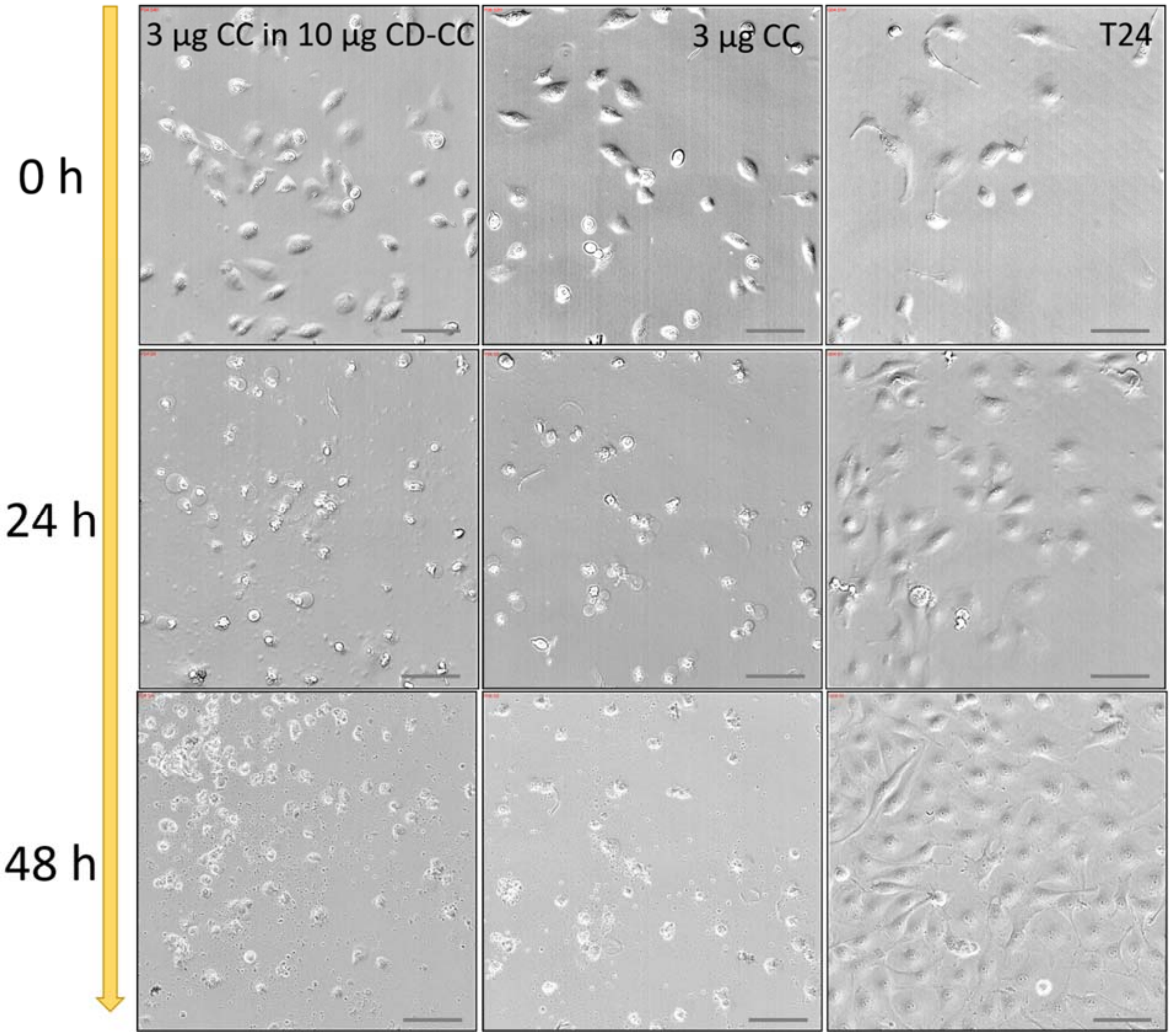

Figure S21. Bright-field images of T24 cells directly after medium exchange (3 h incubation) and after 24 h and 48 h post-incubation. Left column: Incubation with 3 µg CC in 10 µg CD-CC-NP/well. Middle column: incubation with 3 µg CC-NP/well. Right column: untreated T24 cells. Scale bar = 100 µm.

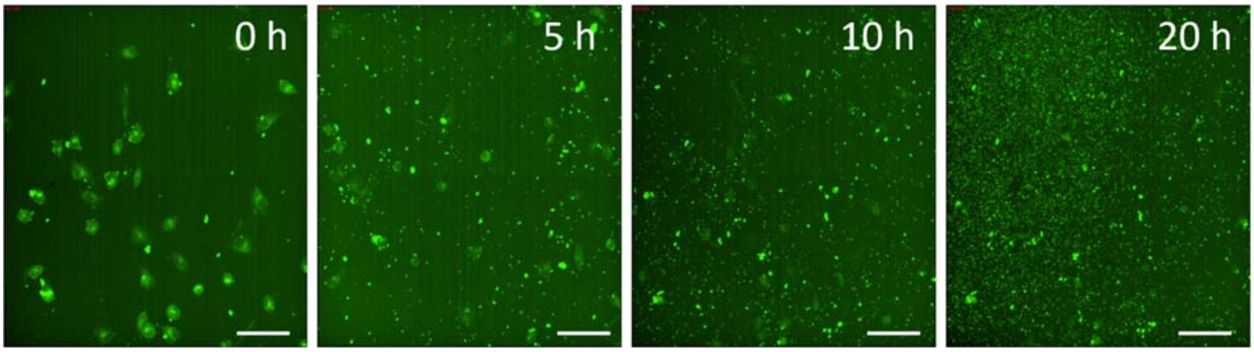

Figure S22. Exocytosis in T24 cells. GFP channel images (green = curcumin) of T24 cells exposed for 3 h to the sample 3/10 µg CD-CC-NP (3 µg CC in 10 µg CD-CC/well, as in Figure S19, left column) showing a time-dependent evolution of expelled fluorescing particles with increasing post-incubation time, indicating exocytosis of cell organelles. Scale bar: 100 µm.

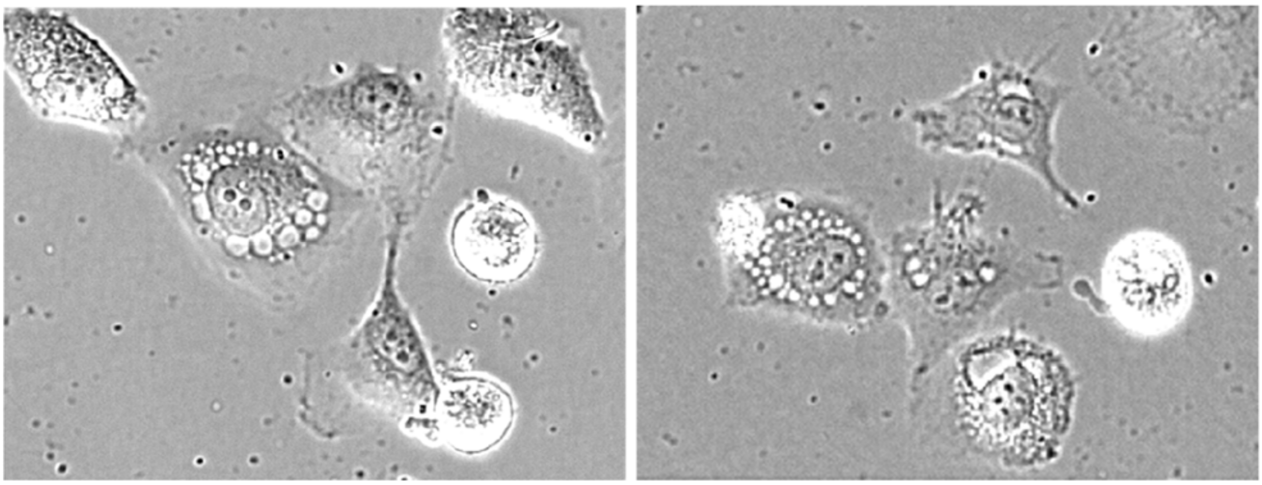

Figure S23. Examples for vacuolization in T24 cells after 1 h incubation and 24 h post-incubation with CD-CC-NP containing 1.8 µg curcumin/well (CD:CC 1:3)

34. Curcumin Delivery to MDA MB-231 Cells.

The impact of curcumin on this cell line is limited when the incubation time of 1 h and 25 h post-incubation time were applied. A CCK-8 endpoint analysis did not show any reduction of cell viability under these conditions (Figure S24a,c,e). Applying image analysis by cell counting indicated some retardation of cell growth when CD-CC-NP were applied at maximum concentrations (b, light blue line; see Figure S24).

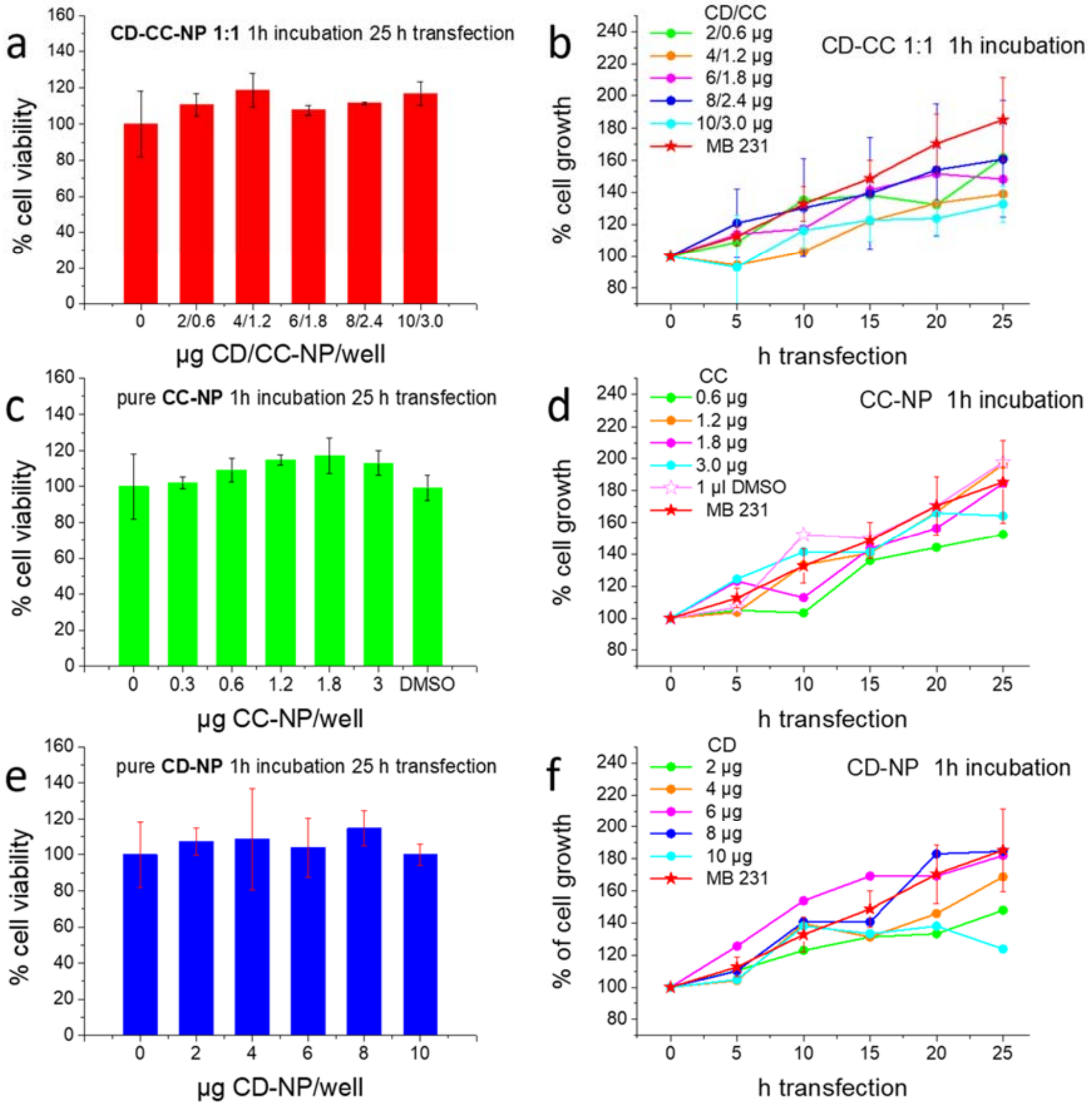

Figure S24. MDA MB-231 incubated for 1 h, assayed with CCK-8 after 25 h post-incubation (**a**,**c**,**d**) and growth curves (**b**,**d**,**f**), right column. a,b treated with CD-CC-NP, c,d treated with DMSO-derived pure CC-NP and e,f treated with pure CD-NP.

When MDA MB-231 cells were incubated for 1 h and followed over the next 25 h post-incubation time after medium exchange, we observed an arrest of cell growth with CD-CC-NP (Figure S25, left columns) but not when exposed to the same concentration of curcumin as CC-NP (Figure S25, right columns). Cells contracted initially, obscuring a distinction of live/dead cells, but recovered after prolonged time as shown in the right column. Exocytosis is also recognized here upon exposure to CD-CC-NP (left column, GFP channel). Further, we note a stronger and longer-lasting fluorescence of CC when contained in CD-NP.

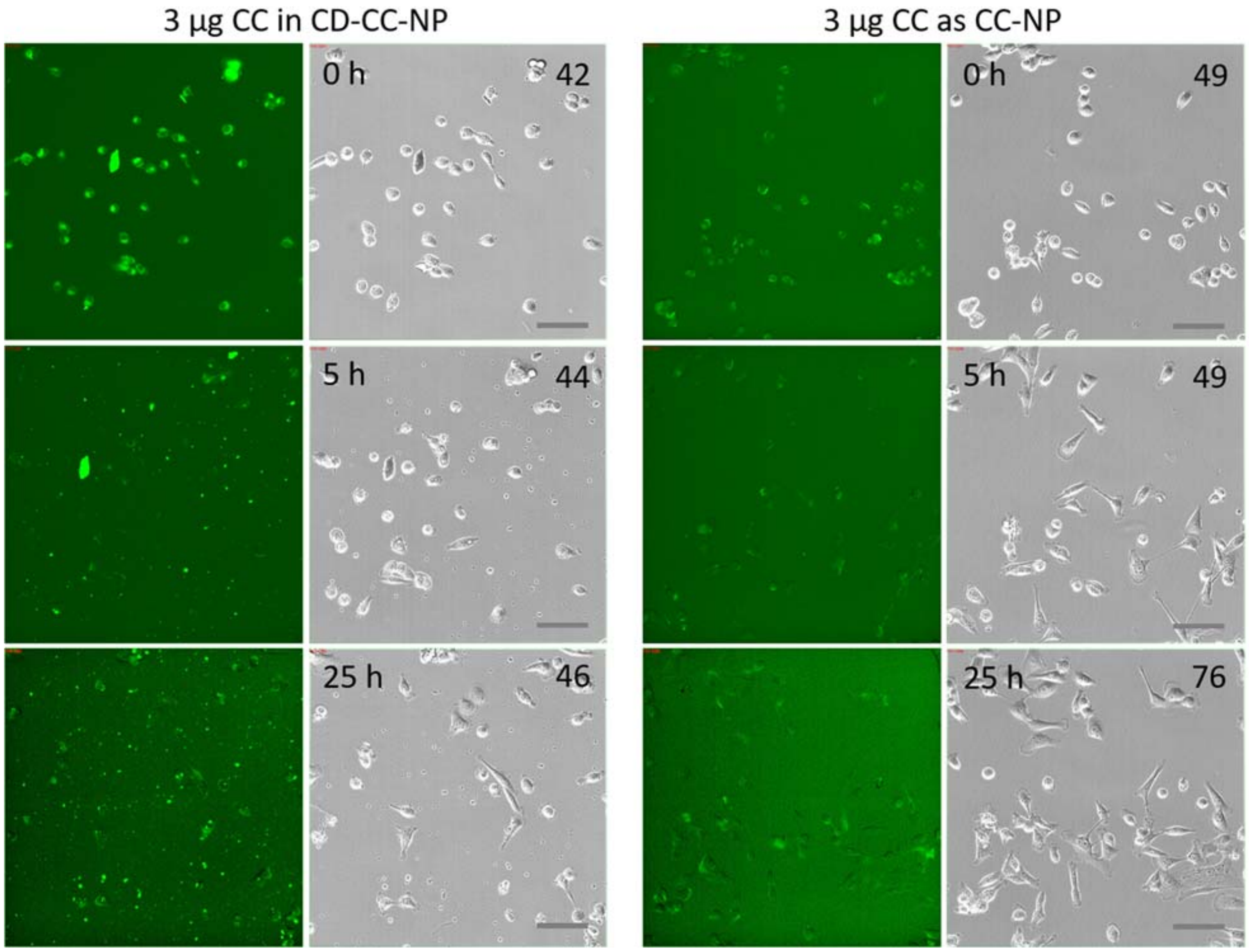

Figure S25. Temporal evolution of MDA MB-231 cells during 25 h post-incubation time after 1 h incubation with 3 µg curcumin/well and subsequent medium exchange. Left columns: curcumin delivered in CD-CC-NP, right column: delivered as CC-NP. GFP channel and transmitted light images of all cells were taken under identical conditions in the same well. The number of cells is indicated in the upper right corner. Scale bar = 100 µm.

35. Curcumin Delivery to MCF-10A Cells.

Vacuola formation in MCF-10A cells was observed only in 2 wells of all experiments performed in this cell line and here only with CC-NP at the highest concentration of 3 μg/well (81 μM) using a 3 h incubation period. After 5 h post-incubation nearly all cells developed large holes, however, even here, we observed a partial recovery of finally about 50 % of the cells after 30 h post-incubation as seen in Figure S26 below.

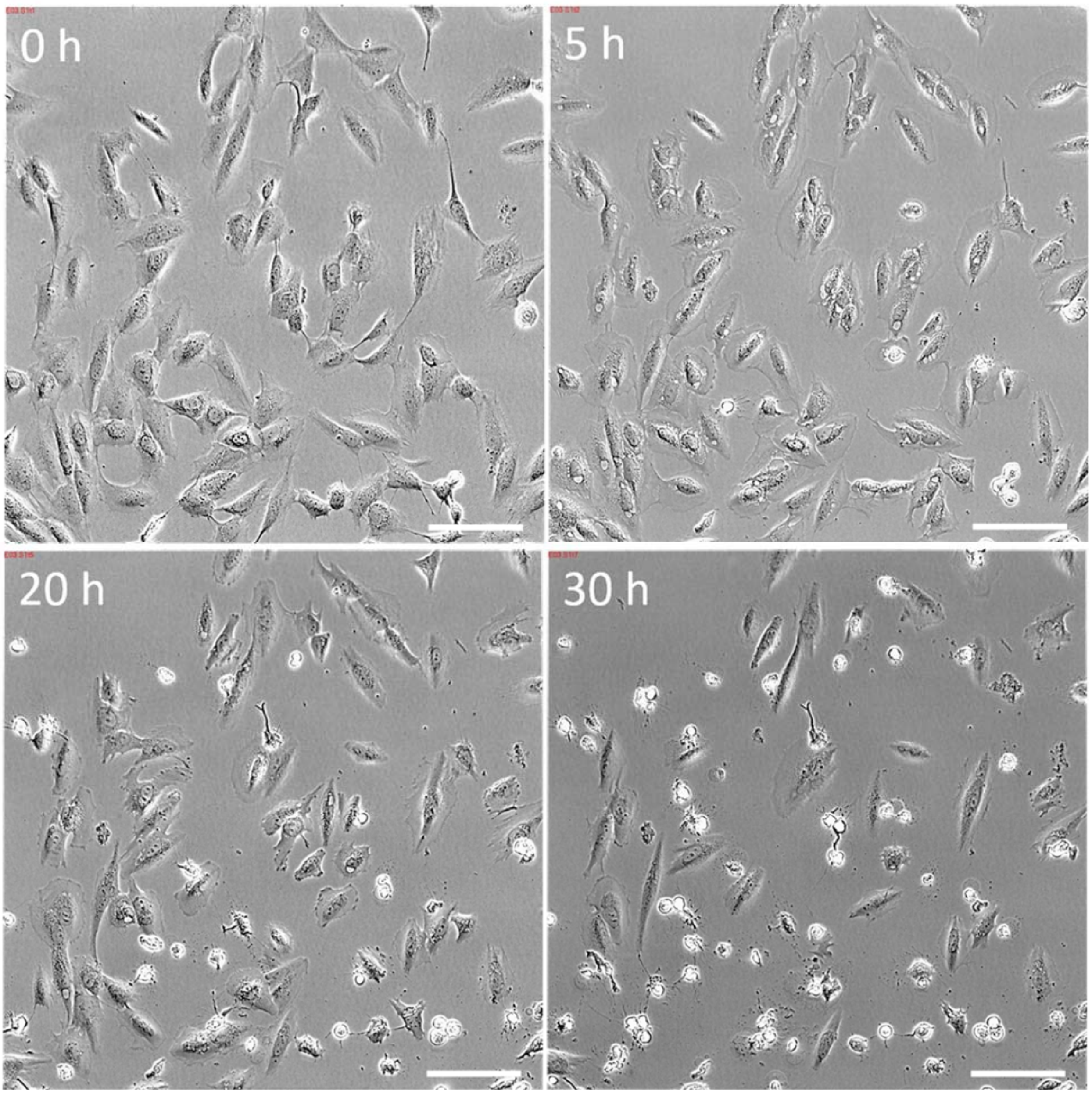

Figure 26. Non-typical vacuola formation in MCF-10A cells after 3 h incubation with 3 μg curcumin nanoparticles CC-NP/well and their partial recovery after 30 h post-incubation. Scale bar = 100 μm.

CCK-8 assays were performed after 48 h post-incubation time on MCF-10A cells that were incubated for 3 h before, showing only minor efficacy for cell killing on this non-cancerous cell line as shown in Figure S27.

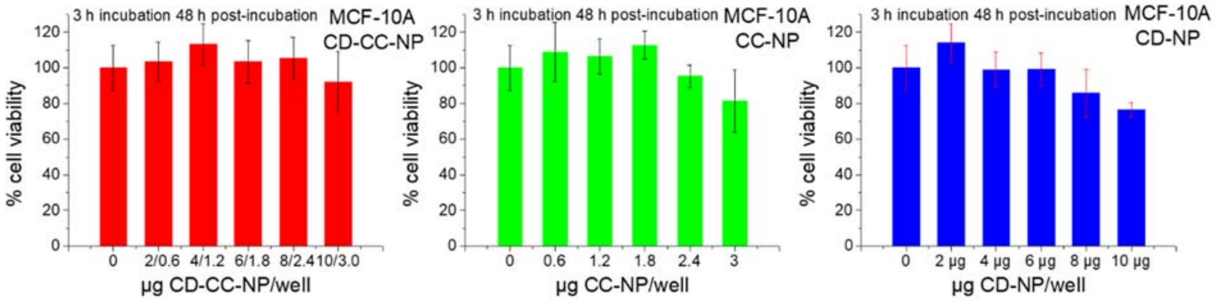

Figure S27. Impact of curcumin on MCF-10A cells. The cells were incubated for 3 h (red: CD-CC-NP with CD:CC = 1:1; green: CC-NP of comparable concentrations to those in the red plot, blue: pure CD-NP of comparable concentrations to those in the red plot). CCK-8 assays were performed after a medium exchange for cell growth buffer and 48 h additional post-incubation time.

References

[1] Bhatia, N.K.; Kishor, S.; Katyal, N.; Gogoi, P.; Narang, P.; Deep, S. Effect of pH and temperature on conformational equilibria and aggregation behaviour of curcumin in aqueous binary mixtures of ethanol. *RSC Advances* **2016,** *6*, 103275–103288.

[2] Mondal, S.; Ghosh, S.; Moulik, S.P. Stability of curcumin in different solvent and solution media: UV-visible and steady-state fluorescence spectral study. *J. Photochem. Photobiol., B* **2016,** *158*, 212–218.
